# Soup to Tree: The Phylogeny of Beetles Inferred by Mitochondrial Metagenomics of a Bornean Rainforest Sample

**DOI:** 10.1093/molbev/msv111

**Published:** 2015-05-08

**Authors:** Alex Crampton-Platt, Martijn J.T.N. Timmermans, Matthew L. Gimmel, Sujatha Narayanan Kutty, Timothy D. Cockerill, Chey Vun Khen, Alfried P. Vogler

**Affiliations:** ^1^Department of Life Sciences, Natural History Museum, London, United Kingdom; ^2^Department of Genetics, Evolution and Environment, Faculty of Life Sciences, University College London, London, United Kingdom; ^3^Division of Biology, Imperial College London, Silwood Park Campus, Ascot, United Kingdom; ^4^Department of Biology, Faculty of Education, Palacký University, Olomouc, Czech Republic; ^5^Department of Zoology, University of Cambridge, Cambridge, United Kingdom; ^6^Entomology Section, Forest Research Centre, Forestry Department, Sandakan, Sabah, Malaysia

**Keywords:** mitochondrial genomes, tree-of-life, biodiversity, phylogeny, Coleoptera, mitochondrial metagenomics, bulk samples, community ecology, metagenome skimming, Illumina MiSeq

## Abstract

In spite of the growth of molecular ecology, systematics and next-generation sequencing, the discovery and analysis of diversity is not currently integrated with building the tree-of-life. Tropical arthropod ecologists are well placed to accelerate this process if all specimens obtained through mass-trapping, many of which will be new species, could be incorporated routinely into phylogeny reconstruction. Here we test a shotgun sequencing approach, whereby mitochondrial genomes are assembled from complex ecological mixtures through mitochondrial metagenomics, and demonstrate how the approach overcomes many of the taxonomic impediments to the study of biodiversity. DNA from approximately 500 beetle specimens, originating from a single rainforest canopy fogging sample from Borneo, was pooled and shotgun sequenced, followed by de novo assembly of complete and partial mitogenomes for 175 species. The phylogenetic tree obtained from this local sample was highly similar to that from existing mitogenomes selected for global coverage of major lineages of Coleoptera. When all sequences were combined only minor topological changes were induced against this reference set, indicating an increasingly stable estimate of coleopteran phylogeny, while the ecological sample expanded the tip-level representation of several lineages. Robust trees generated from ecological samples now enable an evolutionary framework for ecology. Meanwhile, the inclusion of uncharacterized samples in the tree-of-life rapidly expands taxon and biogeographic representation of lineages without morphological identification. Mitogenomes from shotgun sequencing of unsorted environmental samples and their associated metadata, placed robustly into the phylogenetic tree, constitute novel DNA “superbarcodes” for testing hypotheses regarding global patterns of diversity.

## Introduction

The great majority of species on Earth is yet to be discovered, and even for those species that have been formally described, a phylogenetic placement as the basis for comparative biology is currently impossible in most cases (Cracraft 2002; Wheeler et al. 2012). Hence, Charles Darwin’s vision of a largely complete tree-of-life remains to be realized. Next-generation sequencing (NGS) technologies can arguably change this situation, as genomic data provide new phylogenetic information for better tree support (Faircloth et al. 2012; Lemmon et al. 2012; Letsch et al. 2012; Taylor and Harris 2012; Li et al. 2013). NGS technologies may also greatly increase the number of species that can be subjected to DNA analyses (Santamaria et al. 2012; Gibson et al. 2014). However, as currently implemented, NGS studies of species diversity are based on short reads that are matched to existing reference data from conventional sequencing, such as mitochondrial *cox1* “barcodes” or portions of rRNA genes (Gibson et al. 2014), or they rely on sequence-based operational taxonomic units (OTUs) that are not reconciled with Linnaean species (Ji et al. 2013; Cristescu 2014). Hence, NGS-based biodiversity studies currently do not contribute novel data to the framework of the Linnaean taxonomy and the growing DNA-based system associated with it. Instead, while multilocus and genomic NGS studies generally add more data for each terminal in a tree, they do not usually include newly discovered or uncharacterized taxa. As such, NGS approaches do not currently overcome the evident disconnect between research programs in species discovery (Riedel et al. 2013) and various kinds of phylogenomic analysis (Bernt, Bleidorn, et al. 2013; Maia et al. 2014; Misof et al. 2014).

It is current practice in molecular taxonomy and systematics to target specific taxa for their relevance to particular taxonomic and phylogenetic questions. This taxon-focused approach requires the targeted acquisition of DNA-grade specimens and dedicated collecting campaigns. Frequently the desired specimens have to be isolated from much larger samples of untargeted species obtained by general collecting, but NGS approaches could potentially use the entire “biodiversity soup” (Yu et al. 2012) to incorporate a much larger number of species into the taxonomic process. Equally, ecological studies of many poorly known ecosystems would greatly gain from explicit species identification (e.g., against sequence databases) and phylogenetic placement of the sampled specimens, to supplant the current OTU-based analyses.

Mitochondrial genomes can be assembled readily from short-read sequence data (Besnard et al. 2014), and if sequencing is conducted on a species pool, they may assemble into multiple orthologous sequences corresponding to each species present. This “mitochondrial metagenomics” approach, that is, the assembly of numerous mitochondrial genomes from shotgun sequenced mixtures of specimens, has already been demonstrated in several earlier studies (Timmermans et al. 2010; Zhou et al. 2013; Gillett et al. 2014; Tang et al. 2014). In addition, mitochondrial genomes are increasingly acknowledged as powerful phylogenetic markers for resolving relationships at various hierarchical levels, for example, to study intraordinal (Simon and Hadrys 2013) and intrafamilial (Gillett et al. 2014) relationships of insects, in particular under dense taxon sampling (Cameron 2014).

Here, we explore how mitochondrial metagenomics on mixed assemblages can contribute to build the tree-of-life by shotgun sequencing a mass-trapped sample of tropical biodiversity. Mitochondrial genome assemblies could provide a robust phylogeny for the sampled community and feed into a growing mitochondrial genome tree obtained through independent phylogenetic and ecological studies. In turn, by placing these sequences into a broader phylogeny, uncharacterized species assemblages could be described in a more meaningful way than is possible with existing NGS approaches, as accurate assignation of newly assembled sequences places them into an increasingly complete framework of comparative biology and historical biogeography. We focus on the Coleoptera, the most species-rich insect clade, and a sample from the tropical forest canopy in Borneo, one of the richest ecosystems anywhere, to demonstrate the dual pursuit of the phylogenetic placement of a local sample and the increased representation of the tree of Coleoptera. This single sample of less than 500 specimens contained a large proportion of the known lineages of Coleoptera and, even when analyzed on its own, gives a mostly accurate picture of basal coleopteran relationships.

## New Approaches

We take up the challenge of de novo assembly from a complex, uncharacterized sample as it is encountered in a natural setting, without prior sorting or selection of the individuals included. Variability in the amount of input DNA per species (approximately species biomass × abundance) translates to variability between species in the number of mitochondrial reads available for assembly. This is expected to reduce overall assembly success, exhibited as a tendency toward generating complete mitogenomes for some species and fragmented sequences for others. Sequence contiguity for fragmented species may be improved through the use of different assemblers and by combining their output (Gillett et al. 2014), but the increased complexity of natural samples, particularly from intraspecific variation, complicates the generation of a single non-redundant set of contigs for the sample, as algorithmic differences between assemblers may result in slightly different contigs for the same species. The differences may reflect genuine single nucleotide polymorphisms (SNPs) that are present in the sequenced population, or assembly errors. Ideally reads would be mapped to the assembled contigs to help distinguish between these possibilities; however we find that the alignment of reads derived from complex metagenomic mixtures to multiple orthologous sequences is problematic with current tools and may preclude the use of universal similarity settings across genes and taxa.

The huge amount of primary data generated by the metagenomic approach, and the low expected proportion of mitochondrial data (approximately 0.5%; Zhou et al. 2013; Tang et al. 2014) also raises the need for various simplifying steps, for example, by filtering the mitochondrial targets either at the stage of raw reads or after assembly into contigs. With an increasing database of available mitogenome sequences, this filtering step can be directed toward the focal taxa through similarity searches against these reference data, but the conditions for similarity searches face a trade-off between detection of spurious matches and possible loss of true target sequences if stringency is too great. The variable amount of DNA for the species in the pool inevitably results in uneven assembly success, even where contiguity can be improved through the combination of multiple assemblies. This is problematic for the analysis of mitogenomes in a phylogenetic context because a species may be represented by multiple nonoverlapping contigs corresponding to a single mitogenome. This issue can be avoided by conducting phylogenetic analysis on a matrix centered on the most frequently recovered gene to ensure only orthologous sequences are incorporated. This study addresses some of these methodological issues to successfully generate and characterize a largely complete mitogenome phylogeny for a complex biodiversity sample.

## Results

### Tropical Beetle Assemblage and Mitochondrial Genomes

A total of 477 beetles were sorted from a mixed sample of canopy arthropods obtained from insecticide fogging of a rainforest canopy in Danum Valley, Sabah, Malaysia. Morphospecies assignment based on specimen images recognized 209 species in 34 families, in addition to three species (six specimens) not identifiable at family-level (supplementary table S1, Supplementary Material online). Genomic DNA was extracted destructively from each whole specimen individually and DNA extracts of all specimens were pooled using an equal volume per sample to simulate a bulk extraction from unsorted samples. Two such pools were made for Illumina TruSeq library preparation (average insert sizes approximately 480 and 850 bp). Illumina MiSeq sequencing (250 bp, paired-end) yielded 7.9 and 8.1 Gb of raw data for the small and large-insert libraries, respectively. Following removal of sequencing adapters, the proportion of paired reads retained for analysis was 62.96% and 70.78%. After this initial step, the data were further filtered with BLASTn against a database of 245 identified coleopteran mitogenomes (Timmermans MJTN, Barton C, Haran J, Ahrens D, Culverwell L, Ollikainen A, Dodsworth S, Foster PG, Bocak L, Vogler AP, unpublished data), hereafter “MitoDB” (supplementary table S2, Supplementary Material online), at *E* ≤ 1e-5, to retain only “mitochondrial-like” reads for analysis, for a total of 9.91% and 11.24% in the short- and long-insert libraries, respectively. A conservative estimate of the proportion of true mitochondrial reads was obtained by requiring long BLASTn alignments (≥100 bp, *E* ≤ 1e-5) for both reads of each pair to the MitoDB. This indicated that approximately 1.12% (480 bp) and 1.43% (850 bp) were sufficiently similar to the MitoDB sequences to be confidently classed as mitochondrial (table 1).

**Table 1.** Number of Read Pairs after Various Filtering Steps.

	Short Insert Library	Long Insert Library	Total
Raw reads	16,996,158	16,898,216	33,894,374
Adapters removed	10,701,469	11,961,260	22,662,729
BLAST filtered^a^	1,060,340	1,344,092	2,404,432
BLAST filtered + QC^b^	846,156	1,260,119	2,106,275
Estimated mitochondrial^c^	119,647	171,431	291,078

^a^Mitochondrial-like reads used for Celera Assembler.

^b^Mitochondrial-like reads used for IDBA-UD.

^c^Estimate requiring both reads per pair to align to a mitochondrial genome sequence (1e-5, ≥100 bp).

De novo assembly of mitochondrial-like reads was undertaken with Celera Assembler (CA) (Myers et al. 2000) and IDBA-UD (Peng et al. 2012), after appropriate preprocessing (Materials and Methods). The assembly statistics were broadly similar, although CA obtained contigs of higher N50 and mean sequence lengths despite a greater size range and total assembly length (table 2). The coverage reported by CA was also higher than that estimated for IDBA-UD, although these figures are not directly comparable. Both assemblers produced more mitochondrial contigs of ≥1 kb than there were specimens in the sequenced pool and the assembly statistics for this subset again indicate that the two assemblies were similar (table 2). IDBA-UD assembled more contigs ≥15 kb than CA and slightly more that could be circularized (table 3). This shift toward contigs greater than 15 kb at the expense of 5–15 kb contigs suggests that IDBA-UD finds a longer contiguous sequence for several species. However, this assembler produced a greater number of short contigs, particularly in the 1–3 kb size bracket (supplementary fig. S1, Supplementary Material online). Note that the number of mitochondrial contigs reported is conservative as the threshold used to distinguish true mitochondrial sequences was a ≥1 kb BLASTn alignment to MitoDB (supplementary information S1, Supplementary Material online). This effectively discards mitochondrial contigs less than 1 kb in addition to the non-mitochondrial sequences.

**Table 2.** Assembly Metrics after Contig Generation with Different Assemblers for all Contigs and the Mitochondrial Subset.

Assembly (contigs)	Contigs	Minimum (bp)	Maximum (bp)	Mean (bp)	N50 (bp)	Total Length (bp)	Coverage
Celera (all)	11,120	88	19,685	1,050	1,038	11,677,235	16.59^a^
IDBA-UD (all)	12,588	230	19,201	830	794	10,457,969	4.23^b^
Celera (mt)	498	1,007	19,685	5,616.6	12,804	2,797,049	27.99^c^
IDBA-UD (mt)	564	1,001	19,201	4,760.4	14,490	2,684,874	28.12 ^d^
Non-redundant (mt)	504	1,001	19,382	5,906.8	15,650	2,977,013	27.71^e^

^a^Value given by Celera Assembler for “ContigsOnly.”

^b^Not given by the assembler. Calculated as ((mean input read length × number of reads aligned by IDBA-UD)/total length).

^c^Average value obtained with Qualimap from mapping quality controlled reads to the 498 mitochondrial contigs with SMALT (default parameters except −y 0.98).

^d^Average value obtained with Qualimap from mapping quality controlled reads to the 564 mitochondrial contigs with SMALT (default parameters except −y 0.98).

^e^Average value obtained with Qualimap from mapping quality controlled reads to the 504 mitochondrial contigs with SMALT (default parameters except −y 0.98).

**Table 3.** The Number of Mitochondrial Contigs per Assembly and in the Non-redundant Set for Four Size Classes.

Assembly	1–5 kb	5–10 kb	10–15 kb	≥15 kb (circular)
Celera Assembler	333	64	28	73 (44)
IDBA-UD	424	41	18	81 (49)
Non-redundant set	334	45	18	107 (77)

In order to maximize the number of species recovered and the contiguity of their representative sequence, the two assemblies were combined in Geneious (Kearse et al. 2012) by reassembly (Materials and Methods) to form a non-redundant curated data set. This contained 504 contigs, with a mean of 5,906.8 bp and a maximum length of 19,382 bp. In this final data set, there were 107 contigs longer than 15 kb, of which 77 were circularized (table 3; supplementary fig. S1, Supplementary Material online). To explore the effect of the quality-control step, an additional assembly was obtained from IDBA-UD following more stringent quality-control (Materials and Methods). This reduced the number of reads used in the assembly by 13.9% but decreased assembly contiguity only slightly with a 9% reduction in contigs greater than 15 kb and a complementary increase in contigs of 5–10 kb (supplementary information S2, Supplementary Material online).

Mapping the quality-controlled reads to the final set of non-redundant contigs with widely used software packages gave inconsistent results. In all cases, some bases were not covered by the reads (supplementary table S3, Supplementary Material online; see example contig in supplementary fig. S2, Supplementary Material online). Maximizing coverage values would suggest the SMALT software (https://www.sanger.ac.uk/resources/software/smalt, last accessed May 20, 2015) with default parameters as the optimum choice; however, this program otherwise performs poorly (supplementary table S3, Supplementary Material online). In contrast, using SMALT with a percentage identity threshold of 98% gives a higher quality result at a cost of reduced coverage. BWA-backtrack (Li and Durbin 2009) gave similar results, generally slightly outperforming SMALT (98% identity) except for a higher rate of mismatches. Either of these results could therefore be taken as the best approximation of the coverage; however, each is expected to be conservative. We observed that all programs tested show a pattern of reduced coverage at the terminals of the contigs, often decreasing to zero. For circular mitogenomes at least this is an artifact, as the linearized sequence no longer reflects the start and end of the original contig. Similarly, dips in coverage are observed internally for many contigs (usually in the repetitive, AT-rich control region) under either of the two most plausible mappings. This is likely to be a poor reflection of the true coverage found by the assembler; thus we report the coverage values obtained from SMALT (98% identity) as the least likely to contain spuriously mapped reads, with the caveat that the reported values are expected to be conservative. Overall, 0.7% of bases are not covered by the mapped reads, although 94.6% are covered to at least 3× and 64.9% to at least 10× (supplementary table S3 and fig. S3*a* and *b*, Supplementary Material online). Average coverage values between contigs are highly variable, ranging from 1.6× to 313.1× with a mean of 27.7× (supplementary table S3, Supplementary Material online). This reflects, at least in part, the high variability in input DNA per species; under equimolar pooling for 209 species we would have expected an average coverage of 44× for this amount of data. Plotting average coverage estimates against contig length for the two assemblies and the non-redundant contigs shows that the merging of CA and IDBA-UD increases contiguity of many high coverage partial sequences but not all, indicating that this process could be further optimized (supplementary fig. S3*c–e*, Supplementary Material online).

Putative protein-coding gene (PCG) sequences were identified following tRNA-based fragmentation of the contigs and the mapping of these fragments to a mitogenome reference sequence (Materials and Methods). The PCGs were aligned with transAlign (invertebrate mitochondrial code; Bininda-Emonds 2005). Following the removal of a number of short contigs that contained only partial or poorly aligning gene fragments, the total number of contigs contributing to the PCG alignments was 429. Their length distribution was skewed for short and very long contigs, with 120 contigs containing only a single PCG while 112 contigs contained all 13 PCGs (supplementary fig. S4, Supplementary Material online). In total, 70.32% of the assembled nucleotides were contained within contigs covering at least 11 protein-coding loci, whereas contigs containing three or fewer loci represented just 15.65%.

**Fig. 1.**
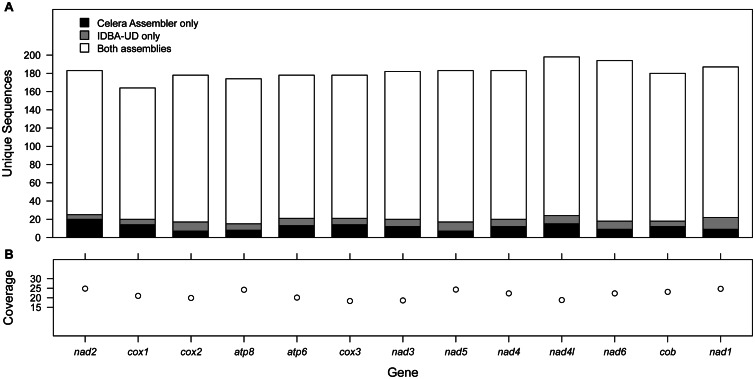
The frequency and coverage of each PCG in the alignments for the non-redundant data set. (*a*) The frequency and source (CA, IDBA-UD, both assemblies) of each unique sequence in the alignment. In all cases, both CA and IDBA-UD assembled the majority of sequences. The inclusion of both assemblies provided a small number of novel sequences for all genes, indicating that neither assembly fully captures the diversity of the sample. (*b*) The coverage by the reads of each base in the alignments is similar between genes and does not explain the variation in frequency in (*a*).

The least frequently recovered gene was *cox1*, present in 164 contigs, while the most frequently recovered was *nad4l*, present in 198 contigs. The rate of recovery of the PCGs and the degree of redundancy between the CA and IDBA-UD assemblies is summarized in figure 1*a*. This shows that the majority of gene sequences was shared between the two assemblies but in all cases, each assembly added a small number of unique sequences. In addition, the number of reads contributing to each base in each alignment (i.e., the average coverage per species per gene) is illustrated in figure 1*b*. This average coverage varies between 18× and 25× in the 13 PCGs, with an overall average of 22.2×.

157 contigs containing the barcode region (*cox1-5′*) were matched to 275 barcode sequences obtained by polymerase chain reaction (PCR) on the individual DNA extracts (see below), successfully linking 124 contigs to morphospecies at an identity threshold of 98% with megablast. Of these, 96 were contigs with 10 or more genes (10+) while 28 were shorter contigs containing between one and eight PCGs (but not *nad4l*). Of the remaining 10+ contigs (28 of 124), 25 contained the barcode region but could not be linked to specimens due to the incompleteness of our barcode database (see below), whereas three contigs did not contain *cox1*.

### Community Phylogeny and the Tree of Coleoptera

Phylogenetic analysis was conducted 1) on the 124 contigs of 10+ genes and 2) on a set of *nad4l*-centred contigs containing a further 51 contigs for a total of 175 terminals of greater than 2 kb in length. As *nad4l* is the most highly represented gene in the data set, the latter matrix serves to maximize the number of haplotypes represented by orthologous sequences, that is, avoids multiple unlinked partial contigs derived from a single mitogenome. A further 23 short contigs of less than 2 kb containing *nad4l* were excluded because the sequence information may be insufficient for their phylogenetic placement, but each is expected to represent a unique species. To incorporate novel species from this assemblage into the growing beetle mitochondrial phylogeny, and to examine the effect of taxon sampling on such analyses, we combined the 10+ contigs with 34 (complete; GenBank: September 2013; hereafter “Mito34”) and 240 (partial and complete; Timmermans MJTN, Barton C, Haran J, Ahrens D, Culverwell L Ollikainen A, Dodsworth S, Foster PG, Bocak L, Vogler AP, unpublished data; hereafter “Mito240”) mitogenomes, respectively. Four analyses were run: 1) 10+ contigs with Mito34, 2) 10+ contigs with Mito240, 3) 10+ contigs alone, and 4) nad4l-centered contigs (includes all 10+ contigs).

**Fig. 2.**
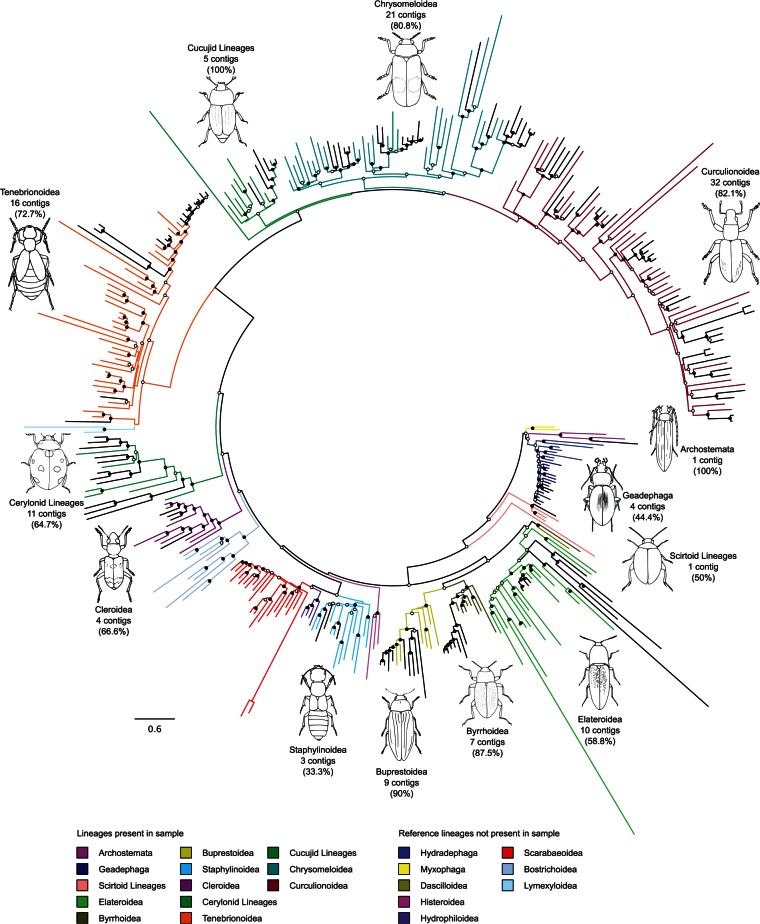
The mitochondrial phylogeny for beetles. The 240 reference mitochondrial genomes are highlighted by superfamily, while 124 contigs contributed by this study, each comprising a minimum of ten PCGs, are shown in black. These novel sequences are distributed throughout the tree, representing most major lineages. The number of novel sequences within each superfamily, and the proportion these represent of the corresponding clade in the community phylogeny (fig. 3*b*), is highlighted. Tree topology and branch lengths are derived from the maximum clade credibility consensus phylogeny from the PhyloBayes analysis. Open circles indicate posterior probabilities ≥0.5, filled circles indicate posterior probabilities ≥0.95.

All trees obtained with PhyloBayes (Lartillot et al. 2009) on the amino acid alignments were rooted with Polyphaga, as the presumed sister group of the other three suborders (Misof et al. 2014). The full tree (10+ contigs combined with Mito240) (fig. 2) was similar to the tree reported based on Mito240 alone, showing: 1) The (Polyphaga ((Myxophaga (Archostemata Adephaga))) basal arrangement of suborders; 2) the branching order at the level of infraorders Scirtoidea, Elateriformia, Staphyliniformia + Scarabaeiformia (paraphyletic due to the separate branch of Histeroidea), Bostrichiformia, and Cucujiformia; 3) the cucujiform relationships of (Cleroidea (Erotylid series (Tenebrionoidea (Cucujoidea (Chrysomeloidea + Curculionoidea)))); 4) the reciprocal monophyly of Curculionoidea and Chrysomeloidea; 5) internal relationships within superfamilies closely matched those of Timmermans et al. (Timmermans MJTN, Barton C, Haran J, Ahrens D, Culverwell L Ollikainen A, Dodsworth S, Foster PG, Bocak L, Vogler AP, unpublished data) and other studies listed therein. The distribution of the 10+ contigs across the tree (fig. 2) indicates a broad representation of major coleopteran lineages in the canopy sample, including three of the four suborders (there was one specimen of Archostemata, whereas the small suborder Myxophaga of mainly semiaquatic and soil dwelling species was not present), and most of the superfamilies in the Coleoptera.

The reliability of phylogenetic assignment relative to the fully identified Mito240 sequences was tested using the 96 10+ contigs that could be linked to specimens and family-level identification through the *cox1* barcodes. We first assessed the taxonomic assignment against Mito34 (supplementary fig. S5, Supplementary Material online). Given the limited sampling of many families by this reduced reference set only 30 contigs were assigned to family (table 4), but the remaining 67 were correctly assigned to higher taxa in the classification (30 to superfamily, 31 to infraorder, 6 to suborder). In the analysis with Mito240 (which includes the Mito34 mitogenomes; fig. 2), 73 contigs were correctly assigned to family and a further 23 contigs to superfamily (table 4), as the much greater taxon coverage permits more detailed assignments relative to multiple representatives of (super)families. In all but one of the latter cases (see supplementary information S3, Supplementary Material online), the failure to make a taxon assignment to family level was due to limited representations of small families (e.g., in Cleroidea).

**Table 4.** Success Rates for Assignation of 96 Baited 10+ Contigs to Higher Taxonomic Ranks^a^.

Taxonomic Rank	Mito34 Topology	Mito240 Topology	BLAST Top Hit (+incorrect)	MEGAN (+incorrect)
Family	30	72	77 (18)	53 (13)
Superfamily	29	23	6	5
Infraorder	31	—	5	9
Suborder	6	1	7	12
Order	—	—	—	10
Subclass	—	—	1	6
Infraclass	—	—	—	1

^a^The table gives the lowest hierarchical level to which a contig was assigned, summed across the 96 contigs in the analysis. Note that the assignations based on the phylogenies were often conservative due to the incompleteness of the reference sets, however in all cases these conservative assignations were correct at the rank at which they were made. In contrast, the majority of BLAST-based assignations were at family level (all but one top hits and most MEGAN assignations) but several were incorrect (shown in brackets), instead requiring identifications to be scored as correct at a given taxonomic rank.

The representation of superfamilies by the contigs in figure 2 was highly variable. This is due both to true differences of species numbers in the canopy sample and to differences in recovery of long mitogenomes. For example, 21 and 32, respectively, of the 124 10+ contigs were placed within the Chrysomeloidea and Curculionoidea, while 3, 1 and 1 contigs were placed within the Staphylinoidea, Scirtoidea and Archostemata (fig. 2). The numbers of contigs per superfamily in this tree, compared with the tree that also includes the shorter nad4l-centered contigs (see below), give some indication of differential sequencing success among lineages. The three 10+ contigs for Staphylinoidea represent one-third of the total number of recovered contigs for this superfamily, that is, the majority of mitogenomes in this lineage was incomplete. In addition, these contigs represent only a small proportion of the estimated 27 morphospecies recognized for this group, whereas in Chrysomeloidea they constitute a mostly complete set of the estimated 25 morphospecies (72 individuals). In some groups mitogenome assembly was virtually complete, for example, four assembled contigs were placed in Erotylidae; all of which were complete, circular and linked to one of the four morphospecies recognized for this group.

**Fig. 3.**
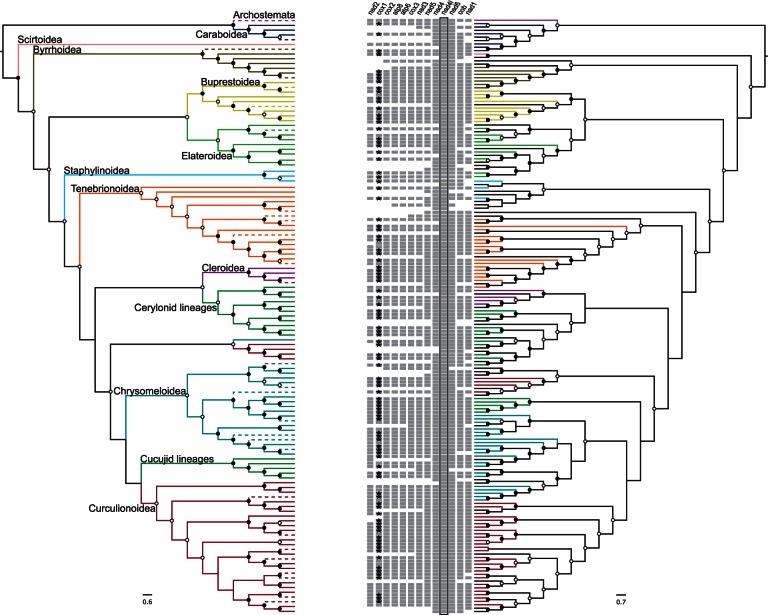
The minimum ten-gene and community phylogenies. (*a*) The minimum ten-gene phylogeny: Maximum clade credibility consensus tree for 124 contigs comprising a minimum of ten PCGs (10+ contigs), colored by superfamily inferred from placement in the phylogenetic tree of beetles (fig. 2). Contigs for which there is a corresponding morphological identification confirming their placement (based on a *cox1* barcode bait sequence) are shown with solid lines, those which either do not contain the barcode region or did not match to any bait sequence are shown with dashed lines. (*b*) The community phylogeny: Maximum clade credibility consensus tree for all 175 contigs longer than 2 kb and containing the most frequently recovered gene, *nad4l*. The colored branches represent the contigs that are in both trees ([*a*] and [*b*]), with the sequences unique to tree (*b*) shown in black. The gene composition of each contig is represented by the presence of gray squares adjacent to the tips, with *nad4l* highlighted by a black box and the presence of a *cox1* barcode sequence indicated by a black star. In both (*a*) and (*b*), open circles indicate posterior probabilities ≥0.5, filled circles indicate posterior probabilities ≥0.95.

The topology from the 10+ contigs alone (fig. 3*a*) was generally similar to the full tree obtained with Mito240. Archostemata was sister to the Caraboidea (suborder Adephaga) and they combined were sister to the Polyphaga. Within Polyphaga, the Scirtoidea was sister to all remaining Polyphaga. Further branches were occupied by a paraphyletic Elateriformia (composed of monophyletic Byrrhoidea, Buprestoidea and Elateroidea), a monophyletic, albeit poorly represented Staphylinoidea, and a monophyletic Cucujiformia, itself composed of the Cleroidea + Erotylid series (formerly considered part of Cucujoidea), Tenebrionoidea, the remaining lineages of Cucujoidea and Chrysomeloidea + Curculionoidea. The latter two superfamilies consisting of the highly species-rich weevils, leaf beetles and longhorns usually grouped as “Phytophaga,” were paraphyletic with respect to each other and this clade also included the Cucujoidea. These problems were alleviated to some extent when the shorter contigs of the *nad4l*-centred matrix were included (fig. 3*b*), as the Chrysomeloidea were monophyletic, but a subset of Curculionoidea (Anthribidae) remained in a distant position from the main clade. Generally, both trees were similar overall and after pruning the short sequences the symmetric difference (Robinson and Foulds 1981; Steel and Penny 1993) was 16 (of a maximum of 242). There were more differences between the Mito240 tree (Timmermans MJTN, Barton C, Haran J, Ahrens D, Culverwell L Ollikainen A, Dodsworth S, Foster PG, Bocak L, Vogler AP, unpublished data) and our tree with Mito240 plus the 10+ contigs, resulting in a symmetric difference of 120 (of a maximum of 474). Differences were affecting basal nodes as well as within-family relationships.

### Identifying Uncharacterized Diversity

Standard PCR-based *cox1* barcodes were obtained for 327 of 477 specimens (68.6% success rate) (supplementary table S1, Supplementary Material online), representing 275 unique haplotypes. When combined with the 157 *cox1-5′* sequences from the assembled mitochondrial contigs, the resulting 326 unique haplotypes were grouped into 232 species-level entities using the GMYC (general mixed Yule coalescent; Pons et al. 2006) method. Out of these putative species, only 113 (48.7%) were shared between the two data sets, 84 (36.2%) were unique to the PCR-based barcodes, and 35 (15.1%) were unique to the mitochondrial contigs. The latter set therefore constituted a substantial proportion that could not be linked to a specimen through the *cox1* baits. All 326 unique barcode haplotypes were searched against the Barcode of Life Database (BOLD) in case a species-level identification was possible. Only one sequence returned a hit, at 98.75% similarity. This was a circular mitogenome (i.e., part of the 10+ set) that had no matching PCR barcode but was assigned to Chrysomeloidea in the tree with Mito34 and Chrysomelidae in the tree with Mito240. The BOLD match was *Liroetiella antennata*, a species of Chrysomelidae (Galerucinae) described from Danum Valley (Sabah, Malaysia) (Bezděk 2013).

In the absence of species-level identifications for the barcodes, we tested the possibility of assigning high-level taxonomy to the mitogenome contigs through the NCBI *nt* database using discontiguous-megablast searches. Based on these analyses, out of 96 identified 10+ contigs with these genes available (93 for *cob*), a total of 32, 77, 54 and 42 contigs were correctly assigned to family based on the *cox1-5′* (barcode), *cox1-3′*, *cox2*, and *cob* loci, respectively, and 82 contigs using any of these genes. The 3′ portion of *cox1* was therefore by far the best performing marker in this analysis, attaining a success rate of 80.2% as compared with 75% for the phylogeny with the Mito240. Taxonomic assignment with MEGAN (Huson et al. 2007), a software that assigns sequences to the nodes in the NCBI Taxonomy database based on Basic Local Alignment Search Tool (BLAST) searches, performed poorly relative to both of these, with only 55.2% of contigs correctly assigned to family (table 4).

## Discussion

### Assembly Success

Samples from highly biodiverse sites can be accumulated rapidly, but the effort for identification and phylogenetic assignment of the recovered specimens frequently is prohibitive. This has resulted in calls for procedures that can avoid the conventional taxonomic process by large-scale DNA sequencing (Blaxter and Floyd 2003; Tautz et al. 2003; Scheffer et al. 2006; Monaghan et al. 2009; Creer et al. 2010; Taylor and Harris 2012). This study demonstrates how complex biodiversity samples can be directly incorporated into a phylogenetic framework by the assembly of short reads from shotgun sequencing of DNA pools. Metagenomic sequencing of individuals encountered in a single fogging event from a Bornean rainforest resulted in a phylogenetic tree from nearly complete mitogenomes of 124 species, corresponding to more than half of all species present. In addition, the 198 *nad4l*-centered contigs provide a nearly complete community set, although some contigs were too short (<2 kb) to be included in the phylogenetic analysis, limiting the community phylogeny to 175 species. Conventional DNA barcoding gave a similar result, recovering sequences for 161 morphospecies and 197 GMYC groups. Given the low rate of barcoding success, possibly due to low DNA quality obtainable from canopy-trapped specimens that were left in situ for up to 24 h, this is clearly an underestimate of the true diversity of the sample. Incorporating the barcode sequences from the contigs we recovered 232 GMYC groups, indicating that the species included in the community phylogeny represent approximately 75% of the total diversity of the sample.

The methodology represents a balance between economical low coverage sequencing of complex samples and the errors and omissions in the assembled contigs that might arise as a result. Greater efficiency of metagenomic sequencing to obtain mitogenome sequences will be key for the wider utility of this methodology, particularly for large-scale community ecology. The estimated proportion of truly mitochondrial reads for both libraries (1.1–1.4%) was more than 2-fold higher than the 0.5% reported from studies on the Illumina HiSeq and shorter insert sizes of the TruSeq libraries of 200 or 250 bp (Zhou et al. 2013; Tang et al. 2014). Although the amount of useable data remains low, this suggests that longer insert sizes may be beneficial for increasing the efficiency of mitochondrial metagenomics, although this was not explicitly tested. Testing two levels of stringency in quality-control settings (High: mean Q30, 5′- and 3′-ends trimmed at Q30; Low: mean Q25, 3′-ends trimmed at Q20) for the reads prior to assembly with IDBA-UD indicated that increased stringency had a relatively small effect on the assembly, although both the number of reads used and overall sequence contiguity reduced slightly (supplementary information S2, Supplementary Material online). Here we used the contigs assembled from the lower stringency settings to maximize the length of the assembled sequences, at the risk of incorporating a small number of erroneous bases, as the impact on the phylogeny would be negligible and mostly lead to slightly increased terminal branch lengths. Filtering the assembled contigs to retain only mitochondrial sequences is complicated by the lack of close reference sequences for the species in the sample. Here we require a BLAST alignment length of 1 kb to a MitoDB sequence, effectively removing long non-mitochondrial contigs but at the cost of mitochondrial contigs less than 1 kb. Thus, we underestimate the number of assembled mitochondrial contigs but the phylogenetic analysis is unaffected due to the 2 kb (with *nad4l*) criterion for inclusion (supplementary information S1, Supplementary Material online). In general we find that assembly quality is difficult to assess, either from general assembly statistics or by mapping reads to the contigs. We suggest that the length distribution of mitochondrial contigs is the most appropriate metric for comparing the suitability of different assemblers for mitochondrial metagenomics, particularly as statistics obtained from mapping are highly dependent on the software and parameters used. The variable mapping quality encountered herein (supplementary table S3 and fig. S2, Supplementary Material online) may have been exacerbated by variable intra- and interspecific sequence divergences in the sample, as read mapping softwares are not optimized to find the best alignment solution for reads derived from the same portion of the genome from multiple species simultaneously. In addition the insect mitogenome is AT-rich, especially in the (repetitive) control region, which may present an additional challenge to both mapping and assembly software (contigs >15 kb generally terminated in this region). Ultimately the phylogenetic trees resulting from the assembled contigs, both with and without the MitoDB sequences, are consistent with existing hypotheses and where we are able to link contigs with a morphological identification their placement is correct. Thus, major errors in assembly are unlikely.

Our results indicate that combining the contigs from multiple assemblies can be beneficial to increase sequence contiguity. This is most evident from the increase in contigs greater than 15 kb and the number and proportion of those that were circularized (table 3), as well as the reduction in the number of short, high coverage contigs (supplementary fig. S3*c–e*, Supplementary Material online). IDBA-UD produces the larger contig set, but both assemblers contribute unique sequences to the alignments indicating that the inclusion of both is beneficial (fig. 1*a*). Additionally, the number of unique sequences produced with either assembler varied between genes so there is no clear basis to recommend one over the other. Additional study is needed of the behavior of these assemblers and the effect of parameter choice in the presence of variable inter- and intra-specific sequence divergence, particularly with respect to variation in rates of evolution between genes. Here we observe that the most conserved locus, *cox1*, was recovered the least frequently and two of the more variable loci, *nad4l* and *nad6*, were recovered the most frequently. This may indicate that closely related species have been combined to a single contig in the *cox1* region, or that intraspecific diversity has been oversplit into different contigs in the *nad4l*–*nad6* region. This variability in gene recovery does not appear to be related to the average coverage obtained for these alignments (fig. 1*b*), which varies only between 18 and 25×, suggesting that this slight enrichment of *nad4l* and *nad6* results from the assembly, rather than any sequencing bias toward this region.

### Mitochondrial Metagenomics and Phylogeny

It is expected that the tree from a single sample would be highly incomplete with regard to the ecological and geographic diversity globally, yet the stand-alone analysis of this set (fig. 3*b*) recovered most of the expected nodes defining major lineages at the superfamily level and a tree topology resembling that of earlier studies based on extensive sampling guided by taxonomic criteria (Hunt et al. 2007; Bocak et al. 2014). The 10+ contigs were placed correctly relative to a set of 34 complete mitogenomes from GenBank, and the precision of their placement was further improved when set against the larger 240 mitogenome set (table 4). The Borneo set struggled with the recovery of several nodes, for example, at the base of Staphyliniformia (distant position of Histeroidea), Tenebrionoidea (paraphyletic for Lymexyloidea), and Phytophaga (non-monophyly of Curculionoidea and Chrysomeloidea), but these were also difficult to resolve in the standalone Mito240 analysis (Timmermans MJTN, Barton C, Haran J, Ahrens D, Culverwell L, Ollikainen A, Dodsworth S, Foster PG, Bocak L, Vogler AP, unpublished data). Instability nearer the tips (within superfamilies) was not further investigated herein as it would require detailed comparisons within the respective subclades based on external data. However, the key findings are that virtually all long contigs of the bulk mitogenome set were placed correctly with respect to family, superfamily and infraorder levels, and that the higher-level relationships of an entire insect order can be gleaned from a single pool of specimens, even with the skewed taxon sampling dictated by the species occurrence at a single collecting site.

To a large extent, the success of this approach is due to the power of mitochondrial genomes as phylogenetic markers and the recent improvement of methods for phylogenetic reconstruction. The utility of mitochondrial PCGs for inferences of deep (order-level) relationships may be unexpected, but past problems with these markers were encountered mainly at interorder or interphylum levels and in studies with sparse taxon sampling limited by conventional sequencing technology, which presumably exacerbated the effects of great heterogeneity in molecular rates and nucleotide composition (Bernt, Braband, et al. 2013; Cameron 2014). Heterogeneous mixture models implemented in the PhyloBayes software provide a means of extracting the phylogenetic signal despite high levels of saturation (Lartillot et al. 2007). Although tip level relationships remained fluid with the addition of new data, the fact that trees from a local environmental sample and the Mito240 set selected for uniform representation of major lineages largely converge would suggest that basal relationships of Coleoptera inferred from mitogenomes are not sensitive to the precise taxon choice, and that the seamless integration of environmental and phylogenetics data is now possible.

A phylogenetic approach to the description of complex (undescribed) canopy communities goes beyond existing analyses that mainly group species into guilds or functional types whose relative proportions have been used to characterize forest ecosystems (Stork 1987; Hawkins and MacMahon 1989), or simple species counts to assess the effects of disturbance, which are equally unsatisfactory (Lawton et al. 1998). Phylogenetic trees provide 1) a measure of evolutionary diversity associated with a community for estimates of conservation value (Faith 1992) and turnover at the level of lineages and local endemicity (Graham and Fine 2008); 2) the placement of species into particular clades of known character composition for a more reliable, trait-based definition of functional guilds, and more accurate determination of species’ memberships in these guilds; and 3) an alternative to detailed species description and placement of a species into the taxonomic hierarchy based on investigation of close relatives, which is currently impossible for a large proportion of living organisms. For example, the composition of the Borneo canopy community consists of presumed predators (in Carabidae, Staphylinidae, Cleridae), herbivores (Chrysomelidae, Curculionidae, including wood boring Buprestidae, Cerambycidae, and some Curculionidae), semiaquatic shredders (Ptilodactylidae), scavengers (Tenebrionidae), saprophages (Nitidulidae) and aphidophages (Coccinellidae), but these classifications are crude (e.g., Novotny et al. 2010 split the insect herbivores of a Papuan rainforest in 19 guilds) and assignment of any one species to these may be inappropriate without proper placement in relation to well-studied species (Hawkins and MacMahon 1989). As expected from a canopy sample, true aquatic lineages, many ground living predators, and large-bodied groups including Scarabaeiformia were entirely absent from the sample. Phylogenetic placement also refines the taxonomic composition and guild structure of the sample in regard to the total lineage diversity. For example, Chrysomelidae from our sample are composed of a broad range of taxa drawn from all three major lineages of the family (Gómez-Zurita et al. 2007), but also recovers clusters of Galerucinae and Eumolpinae that are diverse in most tropical rainforests. Equally, representatives of Carabidae, Tenebrionidae, Coccinellidae, and Ptilodactylidae are phylogenetically clustered. Although our samples were partly identified to subfamily, the tree-based placement provides more specific estimates of phylogenetic clustering relative to samples from other lineages, including other subfamilies. The analysis therefore defines the lineages of the rainforest canopy, relative to the known taxonomic diversity and potentially in relation to communities from other ecosystems or biogeographic regions if treated with the same sequencing approach. Mitochondrial metagenomics therefore provides a comparative framework for the study of global patterns of arthropod biodiversity directly from bulk specimen samples, while assembling an ever more complete tree-of-life. Although this study only analyzed the Coleoptera, a great diversity of other taxa, in particular wasps and ants, flies and spiders (Hymenoptera, Diptera, Araneae) were presented in the same canopy sample, which could add further major branches of the tree.

### Taxonomic Assignment of Uncharacterized Samples

A key step in the evolutionary analysis of biodiversity is the appropriate assignment of a species to a clade or higher-level taxonomic entity. Ideally all contigs would be linked to a species-level identification through the *cox1* barcode, yet in all but one instance no close matches were available in BOLD. In the face of the current incompleteness in barcode coverage at the species level, secure family-level identifications are crucial for describing the taxonomic composition of the sample and understanding the evolutionary relationships. We tested the possibility of assigning contigs to family using the BLAST top hit, MEGAN, and the phylogeny (table 4). The success rate in the former was high for the *cox1-3′* locus that is well represented in Coleoptera, at 80% of verifiable cases, but was below 60% for the other three assessed loci, including the *cox1-*5′ barcode fragment with an error rate of nearly 70%. MEGAN provided accurate assignment in only 55% of cases for the best-represented *cox1-3′* locus. Using BLAST top-hits, 19% of the assignments at family level were incorrect, including three contigs assigned to families known to be absent from the sample (2 contigs assigned to Hydraenidae, 1 to Scarabaeidae). Thus, even the best-performing locus gives a misleading description and the accuracy of any single identification is unknown. Given the high relative abundance of *cox1-3′* sequences for beetles compared with other loci, additional tests would be needed to determine how these findings apply to other insects in mitochondrial metagenomic analyses.

In contrast to the BLAST top-hit, the taxonomic rank at which tree-based assignments were made was highly variable. However, precision increased with database size and in all cases the assignments were correct at the level at which they could be made (table 4). Thus, the phylogeny-based taxonomy assessments can be treated with higher confidence than BLAST-based methods. Note that although the observed success rate for *cox1-3′* BLAST top-hit and the phylogeny were similar herein, the phylogeny included just 240 species whereas a recent assessment of GenBank (Bocak et al. 2014; March 2012) revealed over 7,000 Linnaean species represented by *cox1-3′* that are used by the BLAST-based approach. Given the ease with which mitogenome sequences for vouchered specimens can now be obtained from equimolar pools of either long-range PCR products (Timmermans et al. 2010) or genomic DNA (Gillett et al. 2014), a database of identified sequences with taxon sampling sufficient for family-level phylogenetic placement of otherwise uncharacterized contigs from any arthropod order could be obtained with minimal effort by the scientific community. As seen here, even a single order of magnitude increase in availability (from tens to a few hundreds) of these “superbarcodes” will have a large effect on the taxonomic resolution achievable from a mitochondrial metagenomic approach to arthropod ecology.

## Conclusions

The need for faster species discovery and documentation has led to calls for new approaches in taxonomy (Maddison et al. 2012), with one of the greatest challenges being the creation of a system for organizing organismal information around an evolutionary framework (Parr et al. 2012). The methodological simplicity of a metagenomic approach to uncharacterized communities, combined with the power of mitochondrial genomes for phylogenetics and assembly of the tree-of-life, suggests that the predictive phylogenetic framework required for ecology and biodiversity research is now within reach.

## Materials and Methods

### Sample Collection, Morphological and Barcode Characterization

A sample of arthropods was obtained from insecticide fogging of the rainforest canopy in Danum Valley, Sabah, Malaysia. The sample produced 477 Coleoptera specimens, which were imaged using an SLR camera prior to destructive DNA extraction. Morphological identification of the specimens was undertaken based on visualization of the images. Family level was the minimum target for identification, but more precise Linnaean identifications (e.g., subfamily, genus) were made where possible. Specimens were then assigned to morphospecies within each lowest taxonomic level. All observable features were taken into account when sorting to morphospecies, including body shape and proportions, total length, surface sculpturing, patterning, and coloration. A conservative approach to morphospecies assignment was taken in the case of ambiguously imaged specimens, erring on the side of lumping superficially similar specimens into a single group.

DNA was extracted from each whole specimen individually following the BioSprint 96 protocol for DNA purification from tissues (Qiagen) and eluted to a volume of 200 µl. The DNA extracts were used for PCR amplification and Sanger sequencing of the 5′ portion (barcode region) of *cox1*. Primers CO1F2 and CO1R2 (Baselga et al. 2013) were used in the first instance and failed reactions were repeated using LCO 1490 and HCO 2198 (Folmer et al. 1994) (for details, see supplementary information S4, Supplementary Material online). Sequencing was conducted on an ABI3700 sequencer. Sequences were assembled and edited in Geneious R6.1 (Biomatters Ltd.). A matrix of 648 bp was generated from these sequences, and barcodes were used as “baits” to link the assembled mitogenomes to particular specimens or morphospecies.

For a barcode-based estimate of total species richness, *cox1-5′* sequences derived from PCR and assembled mitogenomes were aligned using MAFFT v7 (Katoh and Standley 2013) and unique haplotypes, defined using a Perl script (http://sourceforge.net/projects/collapsetypes/, last accessed May 20, 2015), were used for phylogenetic reconstruction with RAxML (Stamatakis 2006) (GTRCAT, 100 rapid bootstraps) on the CIPRES Science Gateway (Miller et al. 2010). The resulting tree was made ultrametric using r8s v1.8 (Sanderson 2003) for delimitation of putative species with the GMYC method under the single threshold model (Pons et al. 2006), using the “splits” package (http://r-forge.r-project.org/projects/splits/, last accessed May 20, 2015) in R v3.1.1 (http://www.r-project.org, last accessed May 20, 2015). All unique haplotypes were searched against the BOLD database (http://www.boldsystems.org/index.php/IDS_OpenIdEngine, last accessed May 20, 2015; species level barcode database) to obtain a species-level identification where possible.

### Shotgun Sequencing and Mitochondrial Genome Assembly

Two pools of genomic DNA extracts were prepared using 2 μl per sample to simulate a bulk DNA extraction, and an Illumina TruSeq library was generated from each, with an average insert size of 480 and 850 bp, respectively. Each library was sequenced on a full run of Illumina MiSeq v.2 with 500 cycles and paired-end sequencing (250 bp reads).

A list of all programs and settings used to assemble mitochondrial genome contigs is included in supplementary table S4, Supplementary Material online. A quality assessment of raw FASTQ files for each library was made using FastQC v0.10.1 (www.bioinformatics.babraham.ac.uk/projects/fastqc, last accessed May 20, 2015) prior to the removal of adapter sequences with Trimmomatic v0.30 (ILLUMINACLIP:2:30:10) (Lohse et al. 2012). To simplify the de novo assembly of mitochondrial genomes, the complexity of the data sets was reduced by searching for similarity of the reads (GenBank: SRX392681, SRX392682) against a database of coleopteran mitochondrial genomes (MitoDB), using BLASTn (BLAST v2.2.27+; Altschup et al. 1990) (*E* value 1 e-5; maximum target sequences 1; DUST filtering disabled). MitoDB includes a set of 245 partial and complete mitogenomes covering all major lineages of Coleoptera, including all four established suborders, all 18 superfamilies within Polyphaga and a total 97 families (supplementary table S2, Supplementary Material online). Putative mitochondrial reads were extracted with a Perl script (script 1, Supplementary Material online), allowing for a BLAST hit from either one or both reads per pair.

Assembly of all putative mitochondrial reads from the two libraries was conducted with CA v7.0 (Myers et al. 2000) and IDBA-UD (Peng et al. 2012). For CA, we used largely default settings without any additional preprocessing of the reads (doToggle=1; toggleUnitigLength=500; unitgger=bogart). Prior to assembly with IDBA-UD the reads were passed through a quality control step with Prinseq-lite v0.19.2 (Schmieder and Edwards 2011) to trim low-quality bases and remove short sequences (minimum length 150 bp; trim 3′ bases below Q20; minimum mean quality Q25; no Ns). Pairs where both reads passed quality control were extracted using a cdbfasta pipeline (script 2, Supplementary Material online) and converted to FASTA. Assemblies with IDBA-UD used a similarity threshold of 98% and minimum and maximum *k* values of 80 and 230 bp, respectively. The same settings were used for a second assembly with IDBA-UD using more stringent quality controls to check for the effect of this step on assembly contiguity (minimum length 150 bp; trim 5′ and 3′ bases below Q30; minimum mean quality Q30; no Ns).

Following assembly, the contigs were filtered against MitoDB with BLASTn (*E* value 1e-5; maximum target sequences 1). All contigs with a BLAST alignment length of ≥1 kb (found to be the best balance between correct disposal of non-mitochondrial contigs and the retention of mitochondrial contigs long enough to be incorporated into the final data set, see supplementary information S1, Supplementary Material online, for details) were extracted with cdbfasta and annotated for tRNA sequences using COVE v2.4.4 (Eddy and Durbin 1994) with covariance models of Coleoptera (Timmermans and Vogler 2012) and a Perl script to generate GenBank formatted files (script 3, Supplementary Material online). Annotated contigs over 15 kb were manually checked in Geneious for identical or near-identical overlapping terminal regions and were circularized where possible. Combining multiple assemblies correctly to maximize sequence contiguity while minimizing error requires some flexibility to allow genuine SNPs while discarding assembly errors. Here this is achieved by iterative reassembly and manual curation in Geneious over several steps, as the automated application of thresholds at the whole-contig level will in some cases introduce errors into the consensus sequence for a species, while in other cases con-specific sequences will not be identified as such and will inflate contig-based estimates of species diversity. Initially, the circular contigs from each assembly were assembled at 99% identity to make a non-redundant set against which the linear contigs were then filtered (99% identity) to remove linear sequences from one assembly which were circularized in the other. The remaining linear contigs were then de novo assembled in two steps, merging the most similar contigs first (99% identity), and later incorporating slightly more divergent sequences where appropriate (98% identity). In each step, the assembled contigs were checked manually and edited to resolve any discrepancies between the two assemblies and generate a final set of high-confidence, non-redundant contigs. The observed discrepancies fell mainly into two categories: First, SNPs were observed in several cases, presumably arising from genuine intraspecific variation; second, discrepancies were sometimes observed at the ends of contigs. In general the longer contig was taken to be the correct sequence, otherwise the IDBA-UD contig was preferentially selected due to the application of user-defined quality control parameters and a requirement for 98% identity during assembly. Where indels were observed in one contig relative to the other, the open-reading frame prediction tool in Geneious was used to determine which contig was most likely to be correct, given the expected sizes and arrangements of the PCGs.

We attempted to explore the plausibility of the assembled contigs by mapping the quality-controlled reads to the non-redundant set. Default parameters for paired-end mapping were trialed with variable success for Bowtie 2 (Langmead and Salzberg 2012), BWA-backtrack (Li and Durbin 2009), BWA-MEM (Li 2014), BBMap (http://sourceforge.net/projects/bbmap/, last accessed May 20, 2015), and SMALT (https://www.sanger.ac.uk/resources/software/smalt/, last accessed May 20, 2015), with the latter additionally being used to map reads at 98% identity (−y 0.98). This gave the most plausible result but was highly conservative, thus read mapping could not be used to aid contig verification in the reassembly step. Mapping statistics were obtained with Qualimap (García-Alcalde et al. 2012) and Tablet (Milne et al. 2013) from sorted BAM files (Li et al. 2009).

Inter-tRNA regions of all non-redundant contigs ≥1 kb were extracted using FeatureExtract 1.2 (feature type tRNA; extract intergenic regions) (Wernersson 2005) following the addition of terminal single-base tRNA annotations to ensure the extraction of all non-tRNA portions of the contigs (script 4, Supplementary Material online). These fragments were then mapped with low stringency against a single reference mitochondrial genome (*Tribolium castaneum*, GenBank: NC_003081) in Geneious to sort the fragments into genes (iterate three times; 50% mismatches allowed; search more thoroughly for poorly matching reads). These fragments were extracted gene-by-gene and all fragments covering at least 50% of the length of the corresponding gene in the reference mitogenome were retained.

PCGs were aligned using transAlign v1.2 (translation table 5, invertebrate mitochondrial code; Bininda-Emonds 2005). The resulting nucleotide alignments and their corresponding amino acid alignments were largely accepted but manual curation in Geneious was used to correct a few apparent frameshifts and each gene matrix was trimmed to begin and end with complete triplets. Any fragments with an aligned length of less than 50% of the final alignment for each PCG were discarded following this step.

To assess the impact of combining two assemblies on the rate of recovery of unique gene sequences, the curated sequences from each alignment were searched against a database of inter-tRNA fragments obtained from FeatureExtract for each of the two original assemblies (i.e., prior to reassembly; megablast: Identity 98%, maximum target sequences 1, word size reduced to 5 for *atp8*, *nad4l* and *nad6* [default 28]). Each sequence per alignment was then scored as present in CA, IDBA-UD, or both assemblies. The mean coverage of the sequences in each alignment was also calculated based on megablast searches against a database of the quality-controlled reads (maximum target sequences 1,000,000). For each alignment, the estimated coverage is calculated as the total number of contributing bases divided by the total number of aligned nucleotides (i.e., the number of sequences in the alignment and their length is accounted for). For PCGs longer than 400 bp (all except *atp8*, *nad3,* and *nad4l*), the number of contributing bases is calculated as the sum of the lengths of the BLAST alignment for each read with an alignment length of at least 200 bp at 98% identity. For the three shortest genes, the number of contributing bases is calculated as the sum of the lengths of the BLAST alignment for each read with an alignment identity of 98% and alignment length of ≥50% of the length of the gene in the *Tribolium castaneum* reference genome (GenBank: NC_003081) (i.e., *atp8*: 78 bp; *nad3*: 179 bp; *nad4l*: 144 bp).

### Community Phylogeny and the Phylogeny of Beetles

The alignments were translated into protein sequences in Mesquite v2.75 (http://mesquiteproject.org, last accessed May 20, 2015) and concatenated to form a supermatrix of 3,730 amino acids. We constructed two supermatrices, one requiring the presence of *nad4l* (the most highly represented gene in the data set) and minimum contig length of 2 kb, and another requiring a minimum of ten PCGs per contig (10+ contigs).

To incorporate the most informative novel sequences from this community into the growing mitochondrial phylogeny for beetles, we combined the 10+ contigs with the 245 complete and nearly complete mitogenomes in the MitoDB. The alignments described above were realigned with the equivalent data for the MitoDB, curated, translated and concatenated as previously, for a new data matrix of 3,695 amino acids under a criterion of a minimum representation of ten genes (240 MitoDB sequences met this criterion, hence Mito240). A subset of this supermatrix formed a fourth data set, including the 10+ contigs and a reduced MitoDB set of 34 complete mitogenomes (Mito34; highlighted in supplementary table S2, Supplementary Material online) to assess the impact of low reference taxon sampling on phylogeny-based taxonomic assignment.

Tree searches on the supermatrices were run with PhyloBayes (Lartillot et al. 2009) on the CIPRES Science Gateway (Miller et al. 2010) with an accept value of 0.1, GTR (general time reversible) exchange rates, CAT profile mixture, and constant sites removed. Tree searches were terminated after 1 week and the maximum clade credibility consensus obtained with TreeAnnotator (Drummond et al. 2012) after 50% burn-in. Trees were rooted on the Polyphaga. Tree topologies were compared using the symmetric difference metric (Robinson and Foulds 1981; Steel and Penny 1993) calculated in R with the *phangorn* package (Schliep 2011).

All contig-derived *cox1* sequences were searched against a database of unique PCR-derived *cox1-5′* haplotypes (baits) using megablast (98% identity; maximum target sequences 1) to link the contigs with a morphological identification where possible. A 98% identity threshold was used to allow identification of contigs representing multi-haplotype species even when the assembled haplotype was not represented in the bait database due to PCR or Sanger sequencing failure.

All morphospecies-linked mitogenome sequences from the 10+ contigs were tested for hits of the *cox1*-5′, *cox1*-3′, *cox2* and *cob* fragments in the NCBI *nt* database, using discontiguous-megablast (*E* value 1 e-19, best hit overhang 0.1, best hit score edge 0.1, maximum target sequences 1). The degree of congruence between the top hit in each case and the morphological identification was assessed. For comparison, MEGAN5 (Huson et al. 2007) (MEta Genome ANalyzer) was used to assign the most appropriate taxonomic identification under the minimum coverage heuristic, based on the top 100 discontiguous-megablast hits for the *cox1*-3′ fragment (*E* value 1 e-19, best hit overhang 0.1, best hit score edge 0.1, maximum target sequences 100). These BLAST-based identifications were compared against the assignations suggested by the phylogenetic placement of these contigs in the trees incorporating Mito34 and Mito240. In this case, identifications were to the lowest rank permitted by the tree topology and required monophyly of the contig with the reference sequences.

## Supplementary Material

Supplementary information S1–S4, tables S1–S4, figures S1–S5, and scripts 1–4 are available at *Molecular Biology and Evolution *online (http://www.mbe.oxfordjournals.org/).

Supplementary Data
