# Supplementary material for: Soup to Tree: The Phylogeny of Beetles Inferred by Mitochondrial Metagenomics of a Bornean Rainforest Sample
Source: Mol Biol Evol. 2015 May 8;32(9):2302–16. doi: 10.1093/molbev/msv111 (PMC4540967; doi:10.1093/molbev/msv111)
Supplement: Supplementary Data [file supp_32_9_2302__index.html]

Soup to Tree: The Phylogeny of Beetles Inferred by Mitochondrial Metagenomics of a Bornean Rainforest Sample — Soup to Tree: The Phylogeny of Beetles Inferred by Mitochondrial Metagenomics of a Bornean Rainforest Sample — Supplementary Data 

# Soup to Tree: The Phylogeny of Beetles Inferred by Mitochondrial Metagenomics of a Bornean Rainforest Sample

## Supplementary Data

files

**Files in this Data Supplement:**

- Supplementary Data - pdf file
