# Supplementary material for: Soup to Tree: The Phylogeny of Beetles Inferred by Mitochondrial Metagenomics of a Bornean Rainforest Sample
Source: Mol Biol Evol. 2015 May 8;32(9):2302–16. doi: 10.1093/molbev/msv111 (PMC4540967; doi:10.1093/molbev/msv111)
Supplement: Supplementary Data [file supp_msv111_Crampton-Platt_etal_MBE-14-1160_SupplementaryData.pdf]

Soup to tree: the phylogeny of beetles inferred by mitochondrial metagenomics of a Bornean rainforest sample

Crampton-Platt, A.L., Timmermans, M.J.T.N., Gimmel, M.L., Kutty, S.K., Cockerill, T.D., Chey, V.K., and Vogler, A.P.

Supplementary Data

Table S1 ... 1
Table S2 ... 2
Figure S1 ... 8
Supplementary Information S1 ... 9
Supplementary Information S2 ... 10
Table S3 ... 11
Figure S2 ... 12
Figure S3 ... 13
Figure S4 ... 14
Figure S5 ... 15
Supplementary Information S3 ... 18
Supplementary Information S4 ... 18
Table S4 ... 19
Script 1 ... 20
Script 2 ... 21
Script 3 ... 22
Script 4 ... 24

Table S1. The number of morphospecies and individual specimens therein, and the number of successful *cox1* barcodes available as bait sequences, grouped by family.

Superfamily	Family	Morphospecies	Individuals	PCR success (morphospecies)	PCR success (individuals)
Archostemata	Cupedidae	1	1	0	0
Caraboidea	Carabidae	9	45	7	24
Scirtoidea	Scirtidae	3	3	3	3
Bostrichoidea	Anobiidae	4	5	2	3
Staphylinoidea	Staphylinidae	27	50	21	32
Byrrhoidea	Ptilodactylidae	6	20	6	17
Buprestoidea	Buprestidae	7	14	6	10
Elateroidea	Eucnemidae	6	8	5	7
Elateroidea	Lycidae	4	4	3	3
Elateroidea	Lampyridae	1	2	1	2
Elateroidea	Elateridae	7	13	6	10
Cleroidea	Cleridae	3	5	3	4
Cleroidea	Malachiidae	1	1	0	0
Cleroidea	Prionoceridae	1	1	1	1
Cucujoidea	Coccinellidae	11	21	9	19
Cucujoidea	Corylophidae	4	6	3	4
Cucujoidea	Discolomatidae	4	8	4	6
Tenebrionoidea	Aderidae	4	4	2	2
Tenebrionoidea	Anthicidae	4	6	4	5
Tenebrionoidea	Ciidae	1	1	1	1
Tenebrionoidea	Melandryidae	1	1	0	0
Tenebrionoidea	Mordellidae	5	6	4	4
Tenebrionoidea	Tenebrionidae	9	44	6	30
Tenebrionoidea	Zopheridae	2	2	2	2
Cucujoidea	Nitidulidae	2	2	2	2
Cucujoidea	Erotylidae	4	5	4	5
Chrysomeloidea	Cerambycidae	6	14	4	12
Chrysomeloidea	Chrysomelidae	19	58	14	38
Cucujoidea	Phalacridae	3	4	2	2
Curculionoidea	Anthribidae	9	17	9	15
Curculionoidea	Attelabidae	4	9	4	7
Curculionoidea	Brentidae	2	2	1	1
Curculionoidea	Curculionidae	33	87	19	51
Histeroidea	Histeridae	2	2	1	1
[unknown]	[unknown]	3	6	2	4

Table S2. The mitochondrial genomes used in this study for filtering mitochondrial sequences (n=245), the mitochondrial tree-of-life for beetles (n=240, 10 protein-coding genes required, unused sequences highlighted in grey), and the tree-of-life under reduced reference taxon sampling (n=34, highlighted in blue).

**Suborder/ *Series*/** Superfamily	**Family**	**Subfamily**	**Genus, species**	**Voucher (BMNH)**	**GenBank Accession No.**	**Geographic origin**	**Published by**
**Archostemata**	Cupedidae	Priacminae	*Priacma serrata*	842702	JX412806	USA	
	Ommatidae	Tetraphalerinae	*Tetraphalerus bruchi*		EU877953	Argentina	Sheffield et al. (2008)
**Myxophaga**	Hydroscaphidae	Hydroscaphinae	*Hydroscapha granulum*		AM493667	Italy	Pons et al. (2010)
	Sphaeriusidae	Sphaeriusinae	*Sphaerius* sp.		NC011322	USA	Sheffield et al. (2008)
**Adephaga**							
Geadephaga	Carabidae	Cicindelinae	*Habrodera capensis*	838091	JX412824	South Africa	Timmermans et al. (submitted)
	Carabidae	Harpalinae	*Agonum muelleri*	832916	JX412835	UK	Timmermans et al. (submitted)
	Carabidae	Brachyninae	*Brachinus crepitans*	844234	JX412826	Slovakia	Timmermans et al. (submitted)
	Carabidae	Carabinae	*Calosoma* sp.		GU176340	USA	Song et al. (2010)
	Carabidae	Lebiinae	*Calleida angusticollis*	838156	JX412855	South Africa	Timmermans et al. (submitted)
	Carabidae	Carabinae	*Carabus mirabilissimus*		GQ344500	Korea	Wan et al. (2012)
	Carabidae	Panagaeinae	*Craspedophorus nobilis*	838140	JX412738	South Africa	Timmermans et al. (submitted)
	Carabidae	Pterostichinae	*Cyrtomelis* sp.	838103	JX412794	South Africa	Timmermans et al. (submitted)
	Carabidae	Ctenodactylinae	*Hexagonia terminalis*	838155	JX412768	South Africa	Timmermans et al. (submitted)
	Carabidae	Promecognathinae	*Promecognathus crassus*	743008	JX313665	USA	Timmermans et al. (submitted)
	Carabidae	Trechinae	Trechini gen. sp.	840194	HQ232802	South Africa	Timmermans et al. (2010)
	Trachypachidae	Trachypachinae	*Trachypachus holmbergi*		EU877954	Canada	Sheffield et al. (2008)
Hydradephaga	Aspidytidae	Aspyditinae	*Aspidytes niobe*		AM493668	South Africa	Pons et al. (2010)
	Dytiscidae	Copelatinae	*Copelatus* sp.	679508	JX220995	South Africa	Timmermans et al. (submitted)
	Gyrinidae	Gyrininae	*Macrogyrus oblongus*		NC013249	Australia	Cameron et al. (2010)
	Gyrinidae	Gyrininae	gen. sp.	840223	JX412840	South Africa	Timmermans et al. (submitted)
	Haliplidae	Haliplinae	*Peltodytes quadratus*	794142	JX313685	Madagascar	Timmermans et al. (submitted)
	Haliplidae	Haliplinae	*Haliplus flavicollis*	835590	JX313677	UK	Timmermans et al. (submitted)
	Meruidae	Meruinae	*Meru phylisae*	703458	JX313691	Venezuela	Timmermans et al. (submitted)
	Noteridae		gen. sp.	838166	JX313666	South Africa	Timmermans et al. (submitted)
**Polyphaga**							
Scirtiformia							
Scirtoidea	Clambidae	Clambinae	*Clambus* sp.	840362	JX412725	South Africa	Timmermans et al. (submitted)
	Scirtidae	Scirtinae	*Cyphon* sp.		EU877949	USA	Sheffield et al. (2008)
	Scirtidae	Scirtinae	*Prionocyphon serricornis*	842693	JX412728	Czech Republic	Timmermans et al. (submitted)
	Scirtidae	Scirtinae	gen. sp.	842692	JX412785	Cameroon	Timmermans et al. (submitted)
Staphyliniformia							
Staphylinoidea	Agyrtidae	Agyrtinae	*Agyrtodes labralis*	842916	JX412779	New Zealand	Timmermans et al. (submitted)
	Leiodidae	Cholevinae	*Nargus velox*	833928	JX313674	UK	Timmermans et al. (submitted)
	Silphidae	Silphinae	*Necrophila americana*		GU176343	USA	Song et al. (2010)
	Staphylinidae	Scaphidiinae	*Brachynopus latus*	842925	JX412745	New Zealand	Timmermans et al. (submitted)
	Staphylinidae	Oxytelinae	*Carpelimus* sp.	840291	JX412816	South Africa	Timmermans et al. (submitted)
	Staphylinidae	Aleocharinae	*Chiloporata longitarsis*	724053	JX313678	UK	Timmermans et al. (submitted)
	Staphylinidae	Pselaphinae	*Euplectus karsteni*	729943	KM586245	UK	Timmermans et al. (submitted)
	Staphylinidae	Pselaphinae	*Tychus niger*	742905	KM586244	UK	Timmermans et al. (submitted)
	Staphylinidae	Habrocerinae	*Habrocerus capillaricornis*	844248	JX412795	UK	Timmermans et al. (submitted)
	Staphylinidae	Omaliinae	*Omalium rivulare*	844244	JX412759	UK	Timmermans et al. (submitted)
	Staphylinidae	Aleocharinae	*Oxypoda opaca*	844246	JX412751	UK	Timmermans et al. (submitted)

	Staphylinidae	Oxytelinae	*Oxytelus* sp.	844247	JX412796	UK	Timmermans et al. (submitted)
	Staphylinidae	Paederinae	*Hypomedon debilicornis*	833953	JX313672	UK	Timmermans et al. (submitted)
Hydrophiloidea	Histeridae	Abraeinae	*Abraeus globosus*	844242	JX313690	UK	Timmermans et al. (submitted)
	Histeridae	Saprininae	*Euspilotus* sp.		GU176344	USA	Song et al. (2010)
	Histeridae	Dendrophilinae	*Paromalus flavicornis*	844243	JX412766	UK	Timmermans et al. (submitted)
	Hydrophilidae	Hydrophilinae	*Amphiops globus*	838127	JX412726	South Africa	Timmermans et al. (submitted)
	Hydrophilidae	Hydrophilinae	*Sternolophus* sp.	838163	JX412800	South Africa	Timmermans et al. (submitted)
	Hydrophilidae	Hydrophilinae	*Tropisternus* sp.		GU176339	USA	Song et al. (2010)
	Hydrophilidae	Hydrochinae	*Hydrochus capensis*	840193	HQ232801	South Africa	Timmermans et al. (2010)
Scarabaeiformia							
Scarabaeoidea	Geotrupidae	Bolboceratinae	*Bolboceratex* sp.	838179	JX412746	South Africa	Timmermans et al. (submitted)
	Geotrupidae	Geotrupinae	*Anaplotrupes stercorosus*	843249	JX412838	Germany	Timmermans et al. (submitted)
	Glaphyridae	Glaphyrinae	*Glaphyrus comosus*	843250	JX313673	Israel	Timmermans et al. (submitted)
	Glaresidae	Glaresinae	*Glaresis* sp.	843266	JX412819	South Africa	Timmermans et al. (submitted)
	Hybosoridae	Ceratocanthinae	*Ceratocanthus* sp.	843270	JX412772	Namibia	Timmermans et al. (submitted)
	Hybosoridae	Hybosorinae	*Hybosorus* sp.	838175	JX412828	South Africa	Timmermans et al. (submitted)
	Lucanidae	Lucaninae	*Dorcus parallelopipedus*	843271	JX412841	Czech Republic	Timmermans et al. (submitted)
	Lucanidae		gen. sp.	857087	JX313682	China	Timmermans et al. (submitted)
	Lucanidae	Lucaninae	*Lucanus mazama*		FJ613419	Not available	Sheffield et al. (2009)
	Lucanidae	Lucaninae	*Nigidius* sp.	843262	JX412771	South Africa	Timmermans et al. (submitted)
	Passalidae	Passalinae	gen. sp.	843267	JX412836	Peru	Timmermans et al. (submitted)
	Passalidae	Passalinae	gen. sp.	899706	JX412812	Belize	Timmermans et al. (submitted)
	Scarabaeidae	Melolonthini	*Schizonycha* sp.	843259	JX412739	South Africa	Timmermans et al. (submitted)
	Scarabaeidae	Aphodiini	*Aphodius* sp.	843252	JX412729	South Africa	Timmermans et al. (submitted)
	Scarabaeidae	Melolonthinae	*Astenopholis* sp.	838182	JX412755	South Africa	Timmermans et al. (submitted)
	Scarabaeidae	Dynastinae	*Cyphonistes vallatus*	843257	JX412731	South Africa	Timmermans et al. (submitted)
	Scarabaeidae	Cetoniini	*Leucoscelis* sp.	843253	JX412740	South Africa	Timmermans et al. (submitted)
	Scarabaeidae	Trichini	*Myoderma* sp.	843255	JX412847	South Africa	Timmermans et al. (submitted)
	Scarabaeidae	Sericini	*Pleophylla* sp.	843251	JX412736	South Africa	Timmermans et al. (submitted)
	Scarabaeidae	Anomalini	*Popillia* sp.	843256	JX412777	South Africa	Timmermans et al. (submitted)
	Scarabaeidae	Melolonthinae	*Rhopaea magnicornis*		NC013252	Australia	Cameron et al. (2010)
	Scarabaeidae	Scarabaeinae	*Sarophorus* sp	843265	JX412735	South Africa	Timmermans et al. (submitted)
	Scarabaeidae	Anomalini	*Adoretus* sp.	843258	JX412788	South Africa	Timmermans et al. (submitted)
	Trogidae	Troginae	*Trox* sp.	843260	JX412734	South Africa	Timmermans et al. (submitted)
Bostrichiformia							
Bostrichoidea	Bostrichidae	Bostrichinae	*Apatides fortis*		NC013582	Not available	Sheffield et al. (2009)
	Bostrichidae	Bostrichinae	*Sinoxylon* sp.	838164	JX412742	South Africa	Timmermans et al. (submitted)
	Dermestidae	Anthreninae	*Anthrenus* sp.	840208	HQ232808	South Africa	Timmermans et al. (2010)
	Dermestidae	Anthreninae	*Attagenus hottentotus*	838157	JX412837	South Africa	Timmermans et al. (submitted)
	Dermestidae	Dermestinae	*Dermestes peruvianus*	835562	JX313683	UK	Timmermans et al. (submitted)
	Dermestidae		gen. sp.	838145	JX412839	South Africa	Timmermans et al. (submitted)
	Dermestidae	Trinodinae	*Trinodes hirtus*	724082	JX313664	UK	Timmermans et al. (submitted)
	Ptinidae	Anobiinae	gen. sp.	838099	JX412730	South Africa	Timmermans et al. (submitted)
	Ptinidae	Xyletininae	*Xyletinus* sp.	844236	JX313671	Slovakia	Timmermans et al. (submitted)
	Ptinidae	Ptininae	*Ptinus rufipes*	840469	HQ232819	Czech Republic	Timmermans et al. (2010)
Elateriformia							

Dascilloidea	Dascillidae	Dascillinae	*Dascillus cervinus*	833042	JQ034414	UK	Timmermans & Vogler (2012)
Buprestoidea	Buprestidae	Polycestinae	*Acmaeodera* sp.	838158	JX412781	South Africa	Timmermans et al. (submitted)
	Buprestidae	Polycestinae	*Acmaeodera* sp.		NC013580	Not available	Sheffield et al. (2009)
	Buprestidae	Agrilinae	*Agrilus biguttatus*	833027	JX412786	UK	Timmermans et al. (submitted)
	Buprestidae	Agrilinae	*Agrilus* sp.	838102	JX412834	South Africa	Timmermans et al. (submitted)
	Buprestidae	Buprestinae	*Anthaxia* sp.	840209	JX412831	South Africa	Timmermans et al. (submitted)
	Buprestidae	Chrysochroinae	*Chrysochroa fulgidissima*		EU826485	Korea	Hong et al. (2009)
	Buprestidae	Chrysochroinae	*Perotis lugubris*	840447	X412762	Greece	Timmermans et al. (submitted)
Byrrhoidea	Byrrhidae	Byrrhinae	*Byrrhus* sp.	840448	JQ034419	Slovakia	Timmermans & Vogler (2012)
	Elmidae	Mastiginae	gen. sp.	840216	HQ232810	South Africa	Timmermans et al. (2010)
	Elmidae	Elminae	gen. sp.	842675	JX412724	Czech Republic	Timmermans et al. (submitted)
	Elmidae	Elminae	*Elmis aenea*	843150	JX412821	Slovakia	Timmermans et al. (submitted)
	Elmidae	Elminae	*Limnius perrisi*	843148	JX412747	Slovakia	Timmermans et al. (submitted)
	Eulichadidae	Eulichadinae	*Eulichas baeri*	840452	HQ232812	Philippines	Timmermans et al. (2010)
	Heteroceridae	Heterocerinae	*Heterocerus fenestratus*	840449	HQ232811	Slovakia	Timmermans et al. (2010)
	Limnichidae	Limnichinae	*Byrrhinus* sp.	838097	JX412827	South Africa	Timmermans et al. (submitted)
	Limnichidae	Limnichinae	gen. sp.	842697	JQ034416	Indonesia	Timmermans & Vogler (2012)
	Ptilodactylidae	Ptilodactylinae	*Ptilodactyla* sp.	840214	JX412727	South Africa	Timmermans et al. (submitted)
Elateroidea	Artematopodidae	Artematopodinae	*Eurypogon sp.*	842698	JX412809	Japan	Timmermans et al. (submitted)
	Cantharidae	Cantharinae	*Malthodes minimus*	842708	JX412853	Czech Republic	Timmermans et al. (submitted)
	Cantharidae	Cantharinae	*Cantharis pellucida*	840465	HQ232817	Czech Republic	Timmermans et al. (2010)
	Cantharidae	Chauliognathinae	*Chauliognathus opacus*		NC013576	Not available	Sheffield et al. (2009)
	Elateridae	Elaterinae	*Agriotes ustulatus*	842673	JX412737	Czech Republic	Timmermans et al. (submitted)
	Elateridae	Cardiophorinae	*Dicronychus* sp.	844235	JX412848	Slovakia	Timmermans et al. (submitted)
	Drilidae	Drilinae	*Drilus flavescens*	840459	HQ232815	Malta	Timmermans et al. (2010)
	Elateridae	Elaterinae	*Propsephus* sp.	838123	JX412817	South Africa	Timmermans et al. (submitted)
	Elateridae	Agrypninae	*Pyrophorus divergens*		NC009964	Brasil	Arnoldi et al. (2007)
	Eucnemidae	Macraulacinae	*Fornax* sp.	899821	JX412858	Belize	Timmermans et al. (submitted)
	Lampyridae	Ototretinae	*Drilaster* sp.	840462	HQ232816	Japan	Timmermans et al. (2010)
	Lampyridae	Lampyrinae	*Pyrocoelia rufa*		AF452048	Korea	Bae et al. (2004)
	Lycidae	Dictyopterinae	*Dictyoptera aurora*	844238	JX412733	Slovakia	Timmermans et al. (submitted)
	Lycidae	Lycinae	*Merolycus dentipes*	840457	HQ232814	South Africa	Timmermans et al. (2010)
	Omalisidae	Omalisinae	*Omalisus fontisbellaquaei*	840456	JX412744	Slovakia	Timmermans et al. (submitted)
	Omethidae	Omethinae	*Drilonius striatulus*	840464	JX412822	Japan	Timmermans et al. (submitted)
	Phengodidae	Phengodinae	*Mastinocerus* s.l. sp.	900076	JX412758	Belize	Timmermans et al. (submitted)
	Rhagophthalmidae	Rhagophthalminae	gen. sp.	840461	JX412750	Indonesia	Timmermans et al. (submitted)
	Rhagophthalmidae	Rhagophthalminae	*Rhagophthalmus lufengensis*		NC010969	China	Li et al. (2007)
	Rhagophthalmidae	Rhagophthalminae	*Rhagophthalmus ohbai*		NC010964	Japan	Li et al. (2007)
	Throscidae	Throscinae	*Trixagus* sp.	840455	JX412793	Czech Republic	Timmermans et al. (submitted)
Cucujiformia							
Cleroidea	Chaetosomatidae	Chaetosomatinae	*Chaetosoma scaritides*		NC011324	New Zealand	Sheffield *at al.* (2008)
	Melyridae		gen. sp.	838169	JX412765	South Africa	Timmermans et al. (submitted)
	Melyridae		gen. sp.	840183	JX412770	South Africa	Timmermans et al. (submitted)
	Melyridae		*Psilothrix* sp.	840200	JX412801	South Africa	Timmermans et al. (submitted)
	Melyridae	Malachiinae	*Clanoptilus assimilis*	838184	JX412833	Greece	Timmermans et al. (submitted)
	Melyridae	Malachiinae	gen. sp.	838161	JX412799	South Africa	Timmermans et al. (submitted)

	Phloiophilidae	Phloiophilinae	*Phloiophilus edwardsi*	842910	JX412815	UK	Timmermans et al. (submitted)
	Phycosecidae	Phycosecinae	*Phycosecis limbata*	842917	JX412820	New Zealand	Timmermans et al. (submitted)
	Trogossitidae	Trogossitinae	*Temnoscheila virescens*	900004	JX412752	Belize	Timmermans et al. (submitted)
Lymexyloidea	Lymexylidae	Hylecoetinae	*Hyloecetus dermestoides*	840470	HQ232820	Czech Republic	Timmermans et al. (2010)
	Lymexylidae	Melittommatinae	*Melittomma* sp.	838114	JX412776	South Africa	Timmermans et al. (submitted)
Tenebrionoidea	Aderidae	Aderinae	gen. sp.	842678	JX412763	Cameroon	Timmermans et al. (submitted)
	Anthicidae	Anthicinae	*Formicomus* sp.	840182	JX412857	South Africa	Timmermans et al. (submitted)
	Anthicidae	Anthicinae	*Omonadus floralis*	840487	HQ232825	Czech Republic	Timmermans et al. (2010)
	Boridae	Borinae	*Boros schneideri*	840483	HQ232823	Slovakia	Timmermans et al. (2010)
	Ciidae		gen. sp.	838173	JX412846	South Africa	Timmermans et al. (submitted)
	Melandryidae	Melandryinae	*Mikadonius gracilis*	842714	JX412823	Japan	Timmermans et al. (submitted)
	Melandryidae	Melandryidae	*Osphya bipunctata*	835592	JX313675	UK	Timmermans et al. (submitted)
	Meloidae		gen. sp.	840179	HM486073	South Africa	Timmermans et al. (submitted)
	Meloidae	Meloinae	*Mylabris* sp.	838144	JX412732	South Africa	Timmermans et al. (submitted)
	Mordellidae	Mordellinae	*Mordella atrata*		FJ859904	USA	Cameron et al. (2010)
	Mordellidae	Mordellinae	gen. sp.	838118	JX412844	South Africa	Timmermans et al. (submitted)
	Mycteridae	Hemipeplinae	*Hemipeplus* sp.	900019	JX412852	Belize	Timmermans et al. (submitted)
	Mycetophagidae	Mycetophaginae	*Mycet. quadripustulatus*	840485	HQ232824	Slovakia	Timmermans et al. (2010)
	Mycteridae	Lacconotinae	*Stilponotus mexicanus*	899983	JX412811	Belize	Timmermans et al. (submitted)
	Oedemeridae	Oedemerinae	*Ischnomera cyanea*	840494	JX412790	Czech Republic	Timmermans et al. (submitted)
	Oedemeridae	Oedemerinae	*Oedemera virescens*	840491	HQ232826	Czech Republic	Timmermans et al. (2010)
	Prostomidae	Prostominae	*Prostomis* sp.	842709	JX412787	Indonesia	Timmermans et al. (submitted)
	Pyrochroidae	Ischaliinae	*Ischalia* sp.	840493	HQ232827	Laos	Timmermans et al. (2010)
	Scraptiidae	Anaspidinae	gen. sp.	840252	JX412791	South Africa	Timmermans et al. (submitted)
	Scraptiidae	Anaspidinae	*Anaspis* sp.	840206	HQ232806	South Africa	Timmermans et al. (2010)
	Scraptiidae	Anaspidinae	*Anaspis* sp.	838162	JX412856	South Africa	Timmermans et al. (submitted)
	Scraptiidae	Anaspidinae	*Anaspis* sp.	840490	JX412814	Czech Republic	Timmermans et al. (submitted)
	Scraptiidae	Scraptiinae	*Scraptia* sp.	838150	JX412851	South Africa	Timmermans et al. (submitted)
	Scraptiidae	Scraptiinae	*Scraptia* sp.	838154	JX412825	South Africa	Timmermans et al. (submitted)
	Scraptiidae	Scraptiinae	*Scraptia* sp.	840489	JX412798	Indonesia	Timmermans et al. (submitted)
	Tenebrionidae	Lagriinae	*Adelium* sp.		NC013554	Not available	Sheffield et al. (2009)
	Tenebrionidae	Alecullinae	gen. sp.	838142	JX412775	South Africa	Timmermans et al. (submitted)
	Tenebrionidae	Alecullinae	gen. sp.	840222	JX412818	South Africa	Timmermans et al. (submitted)
	Tenebrionidae	Lagriinae	*Chrysolagria* sp.	838101	JX412760	South Africa	Timmermans et al. (submitted)
	Tenebrionidae	Lagriinae	*Eutrapela ruficollis*	840203	HQ232805	South Africa	Timmermans et al. (2010)
	Tenebrionidae	Alecullinae	*Paramarygmus* sp.	838115	JX412808	South Africa	Timmermans et al. (submitted)
	Tenebrionidae	Lagriinae	*Paratenetus cf. tropicalis*	899913	JX412774	Belize	Timmermans et al. (submitted)
	Tenebrionidae	Diaperinae	*Platydema* sp.	838098	JX412842	South Africa	Timmermans et al. (submitted)
	Tenebrionidae	Coelometopinae	*Strongylium suspicax*	838137	JX412780	South Africa	Timmermans et al. (submitted)
	Tenebrionidae	Tenebrioninae	*Tribolium castaneum*		NC003081	USA	Friedrich and Muqim (2003)
Cucujoidea	Coccinellidae	Coccidulinae	*Coccidula rufa*	832905	JX412767	UK	Timmermans et al. (submitted)
	Coccinellidae	Coccinellinae	*Coccinella septempunctata*		JQ321839	Korea	Kim et al. (2012)
	Cerylonidae	Ceryloninae	*Cerylon histeroides*	840476	HQ232821	Slovakia	Timmermans et al. (2010)
	Corylophidae	Corylophinae	*Gloeosoma* sp.	842694	JX412843	Indonesia	Timmermans et al. (submitted)
	Cryptophagidae	Cryptophaginae	*Micrambina* sp.	842921	JX313663	New Zealand	Timmermans et al. (submitted)
	Cucujidae	Cucujinae	*Cucujus clavipes*		GU176341	USA	Song et al. (2010)

	Discolomatidae	Discolomatinae	gen. sp.	842682	JX412748	Philippines	Timmermans et al. (submitted)
	Erotylidae	Xenoscelinae	*Loberonotha olivascens*	842922	JX412784	New Zealand	Timmermans et al. (submitted)
	Erotylidae	Erotylinae	*Tritoma bipustulata*	840479	HQ232822	Czech Republic	Timmermans et al. (2010)
	Endomychidae	Endomychinae	*Endomychus coccineus*	835578	JX313667	UK	Timmermans et al. (submitted)
	Kateretidae	Kateretinae	*Kateretes pedicularius*	724182	JX313689	UK	Timmermans et al. (submitted)
	Latridiidae	Lathridiinae	*Enicmus brevicornis*	834305	JX313681	UK	Timmermans et al. (submitted)
	Nitidulidae	Cryptarchinae	*Glischrochilus hortensis*	832900	JX412778	UK	Timmermans et al. (submitted)
	Priasilphidae	Priasilphinae	*Priasilpha obscura*		EU877952	New Zealand	Sheffield et al. (2008)
	Phalacridae		gen. sp.	840198	HQ232803	South Africa	Timmermans et al. (2010)
	Propalticidae	Propalticinae	*Propalticus* sp.	844251	JX412792	Australia	Timmermans et al. (submitted)
	Sphindidae	Sphindinae	*Sphindus dubius*	833440	JX412803	UK	Timmermans et al. (submitted)
Chrysomeloidea	Cerambycidae	Disteniinae	*Distenia fimbriata*	668016	JX221000	Belize	Timmermans et al. (submitted)
	Cerambycidae	Lepturinae	*Necydalis ulmi*	704364	JX220989	France	Timmermans et al. (submitted)
	Cerambycidae	Lepturinae	*Rhagium mordax*	832872	JX412743	UK	Timmermans et al. (submitted)
	Cerambycidae	Lepturinae	*Stenurella nigra*	704360	JX220998	Spain	Timmermans et al. (submitted)
	Cerambycidae	Lamiinae	*Psacothea hillaris*		NC013070	Korea	Kim et al. (2009)
	Cerambycidae	Lamiinae	*Anoplophora glabripennis*		NC008221	China	An et al. (unpublished)
	Cerambycidae	Lamiinae	gen. sp.	838149	JX412849	South Africa	Timmermans et al. (submitted)
	Cerambycidae	Prioninae	gen. sp.	744987	JX220991	Madagascar	Timmermans et al. (submitted)
	Chrysomelidae	Bruchinae	gen. sp.	838117	JX412832	South Africa	Timmermans et al. (submitted)
	Chrysomelidae	Cassidinae	*Arescus labiatus*	704488	JX220988	French Guiana	Timmermans et al. (submitted)
	Chrysomelidae	Cassidinae	*Imatidium capense*	704496	JX220993	French Guiana	Timmermans et al. (submitted)
	Chrysomelidae	Cassidinae	*Laccoptera ruginosa*	838107	JX412753	South Africa	Timmermans et al. (submitted)
	Chrysomelidae	Chrysomelinae	*Phaedon armoraciae*	704385	JX220992	UK	Timmermans et al. (submitted)
	Chrysomelidae	Chrysomelinae	*Timarcha goettingensis*		JX412789	UK	Timmermans et al. (submitted)
	Chrysomelidae	Chlamysinae	*Exema canadensis*	704405	JX220990	USA	Timmermans et al. (submitted)
	Chrysomelidae	Clytrinae	*Peploptera acromialis*	840215	HQ232809	South Africa	Timmermans et al. (submitted)
	Chrysomelidae	Criocerinae	*Crioceris duodecimpunctata*		NC003372	Canada	Stewart & Beckenbach (2003)
	Chrysomelidae	Criocerinae	*Lema* sp.	838112	JX412769	South Africa	Timmermans et al. (submitted)
	Chrysomelidae	Cryptocephalinae	*Cryptocephalus* sp.	838109	JX412804	South Africa	Timmermans et al. (submitted)
	Chrysomelidae	Donaciinae	*Donacia distincta*	704419	JX220999	Canada	Timmermans et al. (submitted)
	Chrysomelidae	Eumolpinae	*Pseudocolaspis* sp.	838113	JX412756	South Africa	Timmermans et al. (submitted)
	Chrysomelidae	Galerucinae	*Chapuisia ellenbergeri*	838108	JX412757	South Africa	Timmermans et al. (submitted)
	Chrysomelidae	Galerucinae	*Fleutiauxia armata*	846535	JX412797	China	Timmermans et al. (submitted)
	Chrysomelidae	Galerucinae	*Sphenoraria micans*	846596	JX412854	China	Timmermans et al. (submitted)
	Chrysomelidae	Spilopyrinae	*Spilopyra sumptuosa*	704500	JX220997	Australia	Timmermans et al. (submitted)
	Chrysomelidae	*Sphaerothrix* sp.	unrecognized name	838106	JX412783	South Africa	Timmermans et al. (submitted)
	Cerambycidae	Cerambycinae	*Closteromerus claviger*	840202	HQ232804	South Africa	Timmermans et al. (2010)
	Orsodacnidae	Orsodacninae	*Orsodacne lineola*	679237	JX220994	UK	Timmermans et al. (submitted)
	Megalopodidae	Megalopodinae	*Zeugophora* sp.	840207	HQ232807	South Africa	Timmermans et al. (2010)
Curculionoidea	Anthribidae	Anthribinae	*Chirotenon longimanus*	838143	JX412830	South Africa	Timmermans et al. (submitted)
	Anthribidae	Anthiribinae	*Platystomos albinus*	847772	JN163968	France	Haran et al. (2013)
	Anthribidae	Urodontinae	*Urodontus glabratus*	840197	JX412859	South Africa	Timmermans et al. (submitted)
	Attelabidae	Attelabinae	*Apoderus coryli*	847768	JN163966	France	Haran et al. (2013)
	Attelabidae	Rhynchitinae	*Byctiscus populi*	847767	JN163965	France	Haran et al. (2013)
	Attelabidae	Rhynchitinae	*Deporaus betulae*	832883	JN163945	UK	Haran et al. (2013)

	Brentidae	Taphroderinae	*Anisognathus* sp.	838136	JX412805	South Africa	Timmermans et al. (submitted)
	Brentidae	Nanophyinae	*Nanophyes marmoratus*	833894	JN163946	France	Haran et al. (2013)
	Brentidae	Apioninae	*Rhopalapion longirostre*	847770	JN163967	France	Haran et al. (2013)
	Brachyceridae	Brachycerinae	*Brachycerus muricanus*	696973	JN163970	France	Haran et al. (2013)
	Curculionidae	Baridinae	*Melanobaris laticollis*	847750	JN163955	France	Haran et al. (2013)
	Curculionidae	Ceutorhynchinae	*Ceutorhynchus assimilis*	847753	JN163956	France	Haran et al. (2013)
	Curculionidae	Cossoninae	*Brachytemnus porcatus*	847759	JN163960	France	Haran et al. (2013)
	Curculionidae	Cryptorhynchinae	*Kyklioacalles aubei*	847756	JN163957	France	Haran et al. (2013)
	Curculionidae	Curculioninae	*Anthonomus pomorum*	847726	JN163951	France	Haran et al. (2013)
	Curculionidae	Curculioninae	*Cionus olens*	847732	JN163958	France	Haran et al. (2013)
	Curculionidae	Entiminae	*Liphloeus tessulatus*	847713	JN163947	France	Haran et al. (2013)
	Curculionidae	Entiminae	*Naupactus xanthographus*		GU176345	Argentina	Song et al. (2010)
	Curculionidae	Entiminae	gen. sp.	840191	JX412810	South Africa	Timmermans et al. (submitted)
	Curculionidae	Entiminae	*Otiorh. rugosostriatus*	849828	JN163969	France	Haran et al. (2013)
	Curculionidae	Entiminae	*Sciobius* sp.	838105	JX412782	South Africa	Timmermans et al. (submitted)
	Curculionidae	Entiminae	*Stroph. melanogrammum*	847719	JN163949	France	Haran et al. (2013)
	Curculionidae	Entiminae	*Barynotus obscurus*	847720	JN163950	France	Haran et al. (2013)
	Curculionidae	Entiminae	*Sitona lineatus*	847716	JN163948	France	Haran et al. (2013)
	Curculionidae	Hyperinae	*Hypera plantaginis*	847743	JN163953	France	Haran et al. (2013)
	Curculionidae	Lixinae	*Larinus turbinatus*	847741	JN163952	France	Haran et al. (2013)
	Curculionidae	Molytinae	*Hylobius abietis*	847744	JN163954	France	Haran et al. (2013)
	Curculionidae	Platypodinae	*Platypus cylindrus*	847763	JN163963	France	Haran et al. (2013)
	Curculionidae	Scolytinae	*Scolytus* sp.	847762	JN163962	France	Haran et al. (2013)
	Curculionidae	Scolytidae	*Xyleborus perforans*	838172	JX412850	South Africa	Timmermans et al. (submitted)
	Dryophthoridae	Rhynchophorinae	*Sphenophorus* sp.		GU176342	USA	Song et al. (2010)
	Nemonychidae	Cimberidinae	*Doydirhynchus austriacus*	847764	JN163964	France	Haran et al. (2013)

References

An Y., Huang X., Hu X., Wang B., Mastro V.C. (unpublished) The complete mitochondrial genome of the longhorned beetle *Anoplophora glabripennis*.

Arnoldi F.G., Ogoh K., Ohmiya Y., Viviani V.R. 2007. Mitochondrial genome sequence of the Brazilian luminescent click beetle *Pyrophorus divergens* (Coleoptera: Elateridae): mitochondrial genes utility to investigate the evolutionary history of Coleoptera and its bioluminescence. Gene 405: 1-9.

Bae J.S., Kim I., Sohn H.D., Jin B.R. 2004. The mitochondrial genome of the firefly, *Pyrocoelia rufa*: complete DNA sequence, genome organization, and phylogenetic analysis with other insects. Mol. Phylogenet. Evol. 32: 978-985.

Cameron S.L., Sullivan J., Song H., Miller K.B., Whiting M.F. 2010. A mitochondrial genome phylogeny of the Neuropterida (lace-wings, alderflies and snakeflies) and their relationship to the other holometabolous insect orders. Zool. Scr. 38: 575–590.

Friedrich M. and Muqim N. 2003. Sequence and phylogenetic analysis of the complete mitochondrial genome of the flour beetle *Tribolium castanaeum*. Mol. Phylogenet. Evol. 26: 502-512.

Haran J., Timmermans M.J., Vogler A.P. 2013. Mitogenome sequences stabilize the phylogenetics of weevils (Curculionoidea) and establish the monophyly of larval ectophagy Mol. Phylogenet. Evol. 67: 156-166.

Hong M.Y., Jeong H.C., Kim M.J., Jeong H.U., Lee S.H., Kim I. 2009. Complete mitogenome sequence of the jewel beetle, *Chrysochroa fulgidissima* (Coleoptera: Buprestidae). Mitochondr. DNA 20, 46-60.

Kim K.G., Hong M.Y., Kim M.J., Im H.H., Kim M.I., Bae C.H., Seo S.J., Lee S.H., Kim I. 2009. Complete mitochondrial genome sequence of the yellow-spotted long-horned beetle *Psacothea hilaris* (Coleoptera: Cerambycidae) and phylogenetic analysis among coleopteran insects. *Mol. Cells* 27: 429-441.

Kim M.J., Wan X., Kim I. 2012. Complete mitochondrial genome of the seven-spotted lady beetle, *Coccinella septempunctata* (Coleoptera: Coccinellidae) Mitochondr. DNA 23:179-181.

Li X., Ogoh K., Ohba N., Liang X., Ohmiya Y. 2007. Mitochondrial genomes of two luminous beetles, Rhagophthalmus lufengensis and R. ohbai (Arthropoda, Insecta, Coleoptera) Gene 392, 196-205.

Pons J., Ribera I., Bertranpetit J., Balke M. 2010. Nucleotide substitution rates for the full set of mitochondrial protein-coding genes in Coleoptera. Mol. Phylogenet. Evol. 56: 796–807.

Sheffield N.C., Song H., Cameron S.L., Whiting M.F. 2008. A comparative analysis of mitochondrial genomes in Coleoptera (Arthropoda: Insecta) and genome descriptions of six new beetles. Mol. Biol. Evol. 25: 2499-2509.

Sheffield N.C., Song H., Cameron S.L., Whiting M.F. 2009. Nonstationary evolution and compositional heterogeneity in beetle mitochondrial phylogenomics. Syst. Biol. 58, 381-394.

Stewart J.B. and Beckenbach A.T. 2003. Phylogenetic and genomic analysis of the complete mitochondrial DNA sequence of the spotted asparagus beetle *Crioceris duodecimpunctata*. Mol. Phylogenet. Evol. 26: 513-526.

Timmermans M.J., Dodsworth S., Culverwell C.L., Bocak L., Ahrens D., Littlewood D.T., Pons J., Vogler A.P. 2010. Why barcode? High-throughput multiplex sequencing of mitochondrial genomes for molecular systematics. Nucl. Acids Res. 38(21),

Timmermans M.J.T.N. and Vogler A.P. 2012. Phylogenetically informative rearrangements in mitochondrial genomes of Coleoptera, and monophyly of aquatic elateriform beetles (Dryopoidea). Mol. Phylogenet. Evol. 63: 299–304

Timmermans M.J.T.N., Barton C., Haran J., Ahrens D., Culverwell C.L., Ollikainen A., Dodsworth S., Foster P.G., Bocak L., Vogler A.P. Submitted. A robust phylogenetic tree of Coleoptera from mitochondrial genomes affected by compositional heterogeneity.

Wan X., Hong M.Y., Liao A., Kim M.I., Kim K.G., Han Y.S., Kim I. 2012. Complete mitochondrial genome of a carabid beetle, *Damaster mirabilissimus mirabilissimus* (Coleoptera: Carabidae). Entomol. Res. 42: 44-54.

Figure S1. Raw contig length distribution for the Celera Assembler and IDBA-UD assemblies, and the non-redundant set generated by merging these in Geneious. The number of contigs longer than 15 kb that could be circularised in each case is shown within the corresponding circular arrow.

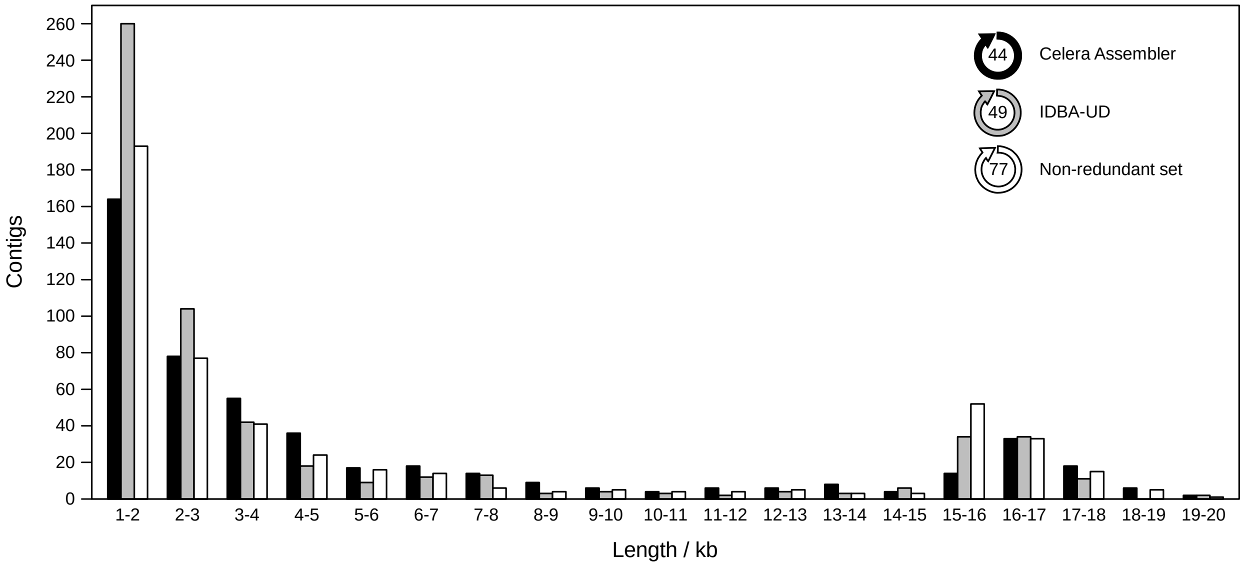

Supplementary Information S1. Choice of BLAST alignment length cut-off when filtering contigs against MitoDB.

The 1 kb threshold was chosen as a good balance between the correct retention of contigs for the inclusion in later analyses, and the loss of shorter contigs that are truly mitochondrial but do not contribute to the final dataset. Determining what is a “true” mitochondrial contig without a closely related reference sequence can be challenging and we aim to include only the most plausible and informative contigs in the final dataset. To illustrate this we present an analysis based on the blastn results for the Celera Assembler contigs against MitoDB. Here we look at the alignment length as a proportion of contig length, with the assumption that true mitochondrial contigs are likely to have proportionately longer hits to the MitoDB sequences than non-mitochondrial contigs. Three different alignment length cut-offs are tested (100 bp; 500 bp; 1 kb). Figure A indicates that a short alignment length cutoff of 100 bp retains large numbers of contigs that are unlikely to be mitochondrial as the BLAST alignments to the database are short relative to their total length (0-40%). From Figure B we can see that those contigs are also, on average, quite short (<3kb). Including these contigs will likely increase downstream processing complexity with minimal gain in aligned data as most, if not all, will be non-mitochondrial. In contrast, Figure A indicates that alignment length cutoffs of 500bp and 1 kb give very similar alignment proportion profiles, except that with a 1 kb cutoff you retain overall a lower number of contigs, especially in the 90-100% aligned category. This suggests that there are a large number of contigs retained by the 500 bp cutoff that have long alignments relative to their lengths (i.e. are probably mitochondrial) but are <1kb in total length. This is reinforced by the low average length of the contigs in this category in Figure B. We can therefore estimate there are at least 250 mitochondrial contigs of 0.5-1kb from this assembly that we have missed from our analysis (this set has a mean length of 713 bp). Simultaneously, the 500 bp cutoff retains two contigs of 9kb with alignment proportions of <10%. BLASTing these on the NCBI website indicates that they are bacterial but there are no highly similar matches. Using a 1 kb cutoff has correctly filtered these out, but at the cost of losing a large number of short contigs. Given that our aim is to retain contigs that can ultimately be included in the phylogenetic analysis, the loss of these short contigs is not problematic for this study as they would not have met the 2 kb (including *nad4l*) criterion.

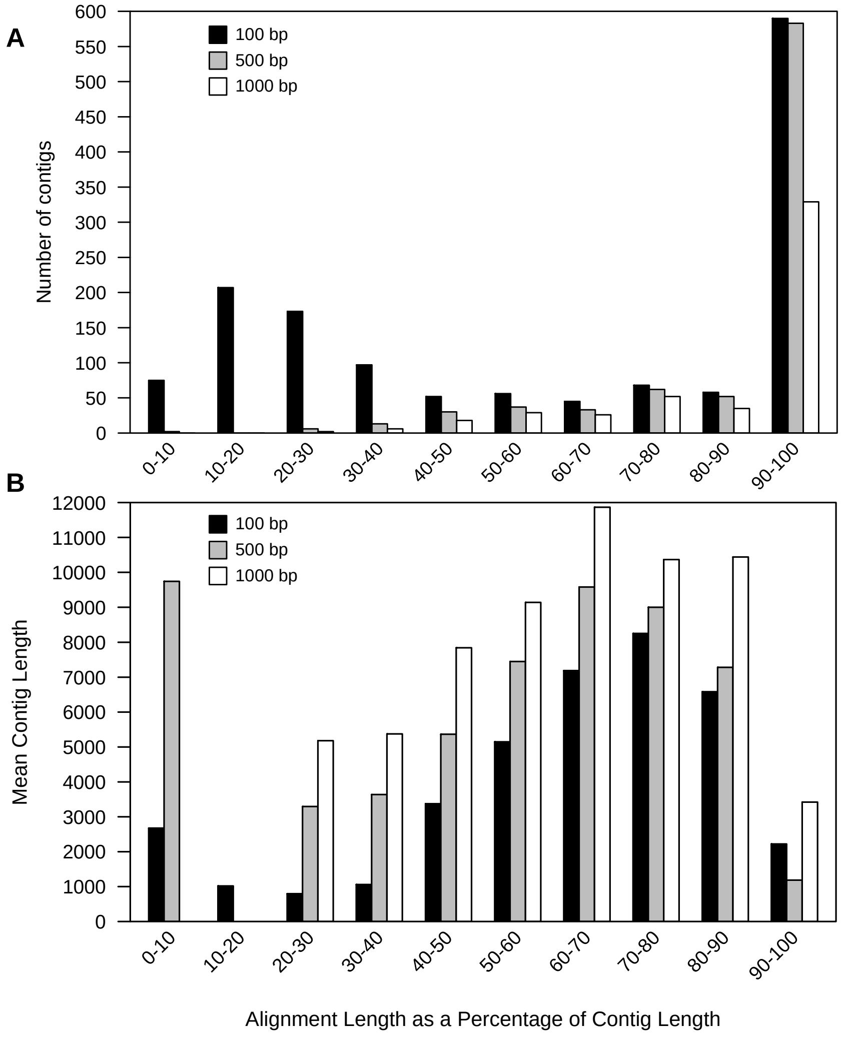

Supplementary Information S2. The effect of quality control on the IDBA-UD assembly.

The IDBA-UD assembly statistics and mitochondrial contig length distributions following two different quality control procedures are outlined in the tables below. In addition, we made comparisons between the circular genomes from the two assemblies. Out of 45 mitogenomes found following QC-2, 39 are >99.9% identical to ones from QC-1. The remaining 6 are circular versions of >15kb linear contigs from QC-1 (at >99% identity i.e. they assemble slightly better under QC-2). 9 of the 10 remaining circular contigs from QC-1 are recovered >10kb at >99% identity under QC-2. The final one was recovered at 100% identity within the PCGs but there are differences in the control region. The contigs from QC-1 were retained for analysis.

Assembly statistics

IDBA-UD Assembly	Contigs	N50	Maximum contig length	Mean contig length	Total assembly length
QC-1[a]	12588	794	19201	830	10457969
QC-2[b]	10851	837	19044	867	9412984

Mitochondrial contig length distribution

IDBA-UD Assembly	1-5 kb	5-10 kb	10-15 kb	>15 kb (circular)
QC-1[a]	424	41	18	81 (49)
QC-2[b]	421	45	22	74 (45)

[a] Prinseq-lite: minimum length 150 bp; trim 3' bases below Q20; minimum mean quality Q25; no Ns; both reads per pair must pass QC. Total number of pairs passing QC: Short insert library 846,156; Long insert library 1,260,119.

[b] Prinseq-lite: minimum length 150 bp; trim 5' and 3' bases below Q30; minimum mean quality Q30; no Ns; both reads per pair must pass QC. Total number of pairs passing QC: Short insert library 690,388; Long insert library 1,131,850.

Table S3. Statistics derived from mapping quality controlled reads to the non-redundant contigs (n=429) with five programs using default settings for paired reads. Additionally SMALT was tested with a requirement for 98% identity under otherwise default parameters.

Program	Minimum mean coverage (s.d.)	Maximum mean coverage (s.d.)	Mean coverage (s.d.)	% bases at 0x	% bases at 3x	% bases at 5x	% bases at 10x	No. contigs where $\sigma > \mu$
**Bowtie**	1.5 (1.0)	351.2 (133.9)	32.3 (49.0)	0.28	96.49	89.81	70.06	6
**BWA aln**	0.8 (0.8)	340.2 (131.1)	30.6 (46.8)	0.54	95.74	88.4	68.24	4
**BWA mem**	3.6 (2.4)	557.4 (3139.1)	43.4 (265.6)	0.09	98.31	93.9	78.24	200
**bbmap**	0.3 (0.5)	389.7 (81.5)	34.9 (51.0)	1.18	95.86	89.89	71.13	24
**SMALT**	3.9 (2.9)	469.9 (1255.8)	57.5 (279.3)	0.08	98.6	95.09	81.82	303
**SMALT (98%)**	1.55 (0.80)	313.1 (126.0)	27.7 (42.5)	0.7	94.59	86.1	64.91	3

Minimum % mismatch	Maximum % mismatch	Mean % mismatch	% GC[b]	Mapping Quality	No. reads mapped	No. reads mapped (proper pairs)	No. reads mapped (singletons)
0.1	6.6	1.2	23.56	32.95	387,045	233,488	26,485
0	3.1	0.5	23.61	48.96	379,632	356,642	10,520
1	8	1.1	20.06	45.05	1,370,236	1,251,539	76,361
0.2	55.9[a]	3.7	23.23	39.19	404,752	362,374	34,632
0.7	15.1	5.5	16.63	35.79	2,151,320	615,390	872,796
0	1.3	0.3	23.63	48.39	330,544	280,428	50,116

[a] Setting maxindel=0 reduces this to 11.7%
[b] GC content of contigs: 23.9%

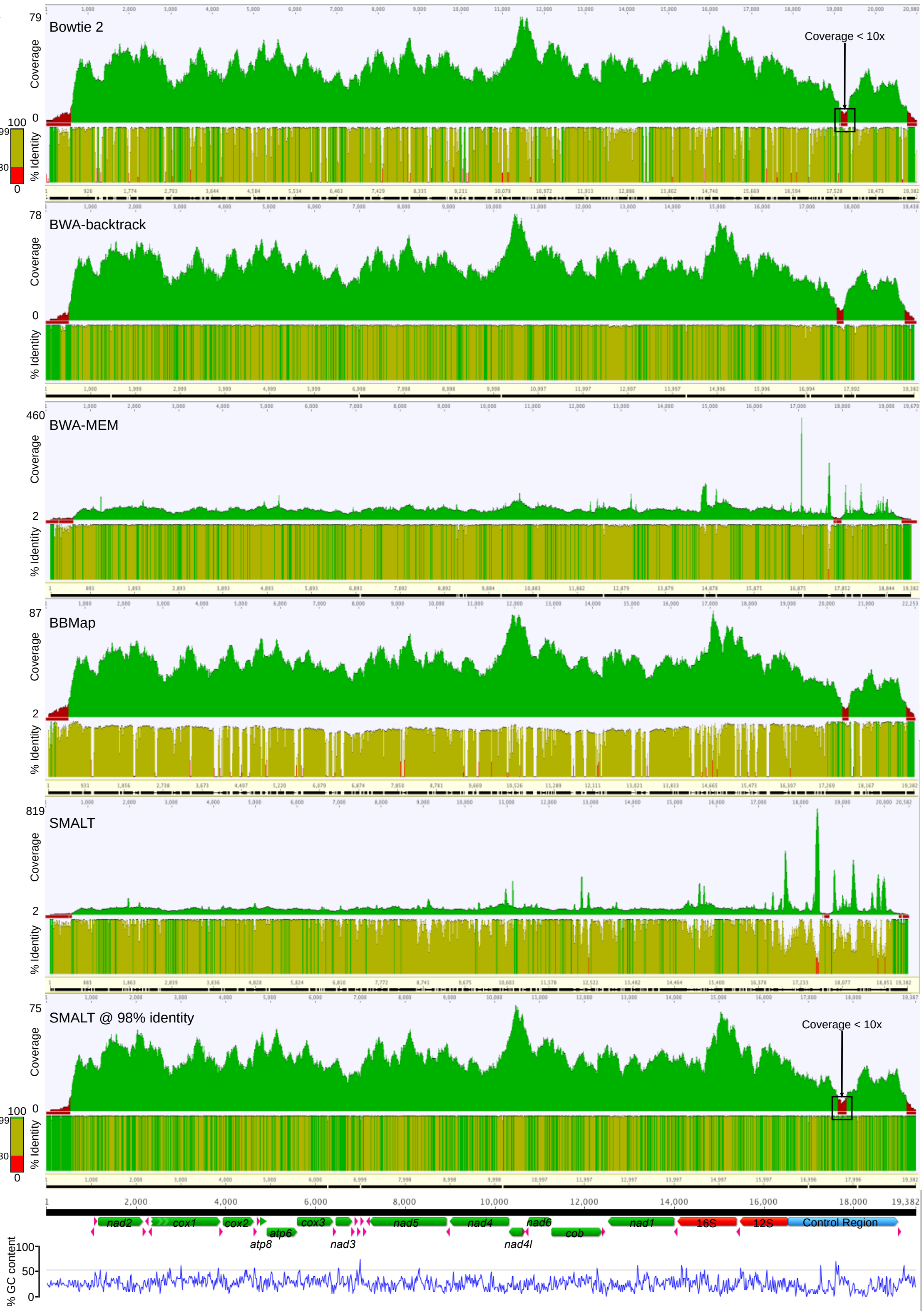

Figure S2. The variability in mapping quality for a single contig. Quality-controlled reads were mapped against all non-redundant contigs. This example shows the coverage and mean pairwise identity of the reads at each position for the longest contig (19,382 bp). All programs introduced gaps into the contig and coverage always decreases at the edges. In all cases there is a region of <10x coverage in the control region. This may indicate misassembly, although both assemblers reconstructed this region identically. BWA-MEM and SMALT map the largest number of reads but the coverage is highly uneven. For the other four examples the coverage maps are similar and more even, although Bowtie 2 and BBMap introduce a large number of gaps into the contig and many positions have low pairwise identity between reads (i.e. many disagreements). BWA-backtrack and SMALT (mapping at 98% identity) give a very similar result and are likely to be the best representation of the assembly, although the number of reads mapped is likely to be conservative.

Software	Mean Coverage (s.d)	No. reads mapped	% Mismatch	No. gaps in contig
**Bowtie 2**	39.60 (12.80)	3240	0.9	1493
**BWA-backtrack**	38.77 (12.75)	3284	0.5	10
**BWA-MEM**	45.06 (22.88)	7001	0.6	49
**BBMap**	42.98 (NaN)	3342	6.3	2773
**SMALT**	52.69 (47.19)	10324	2.6	1057
**SMALT (98%)**	35.36 (12.36)	2884	0.3	5

Figure S3. Coverage plots derived from mapping quality controlled reads with SMALT at 98% identity. A. Percentage of bases in non-redundant contigs for the given coverage (maximum of 50X shown). B. Percentage of bases in non-redundant contigs with at least the given coverage (maximum of 50X shown – 13% of bases have >50X coverge). C. Mean coverage of mitochondrial contigs from the CA assembly plotted against contig length. D. Mean coverage of mitochondrial contigs from the IDBA-UD assembly plotted against contig length. E. Mean coverage of mitochondrial contigs from the non-redundant set plotted against contig length.

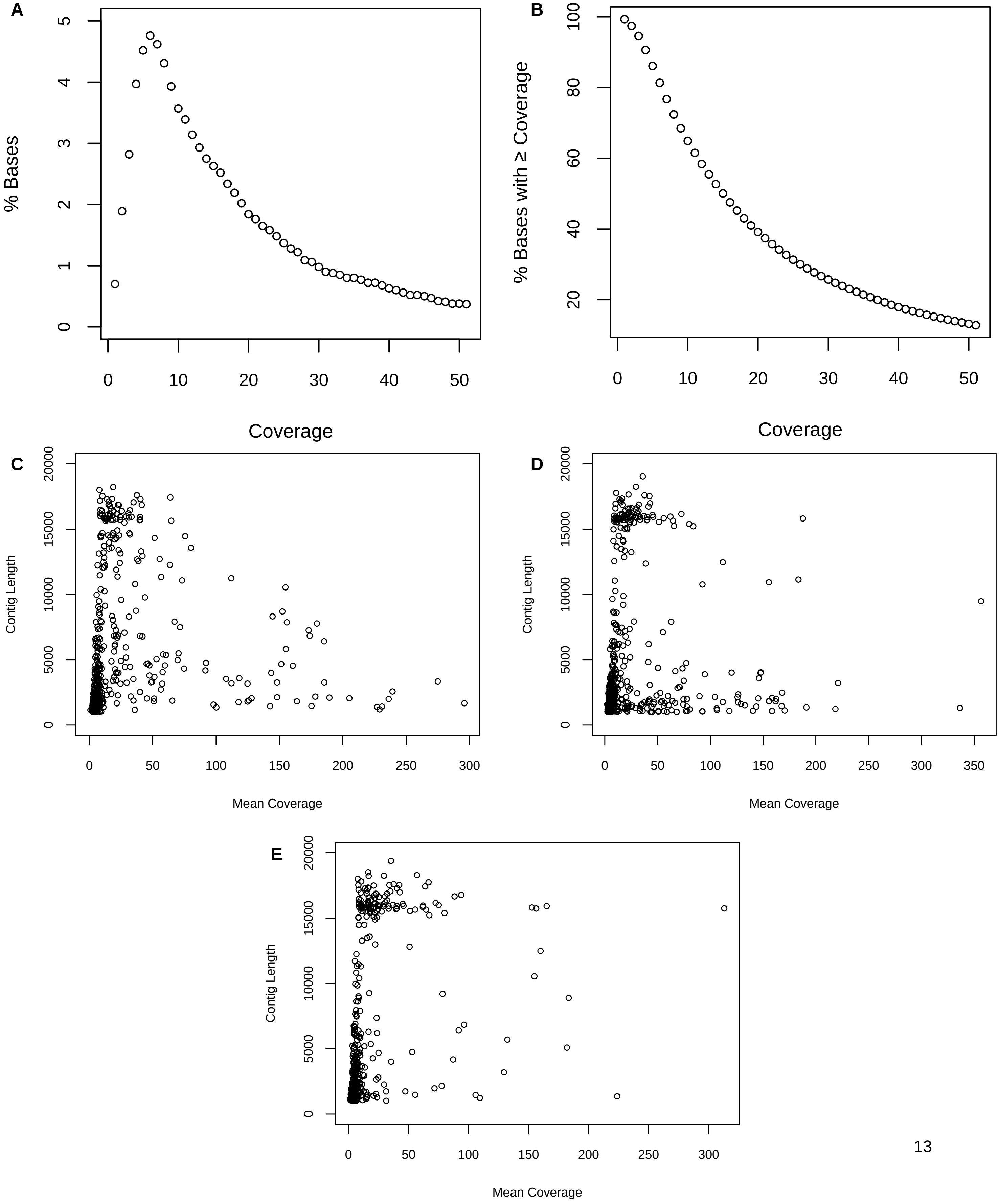

Figure S4. The number of protein-coding genes contributed to the final alignments per contig, for all 429 contigs in the non-redundant set. Approximately the same number of contigs contributed a single gene sequence (120) as the full complement of 13 mitochondrial protein-coding genes (112). Overall, contigs contributing 1-3 protein-coding genes made up 15.65% of the nucleotide matrix whilst those contributing 11-13 made up 70.32%.

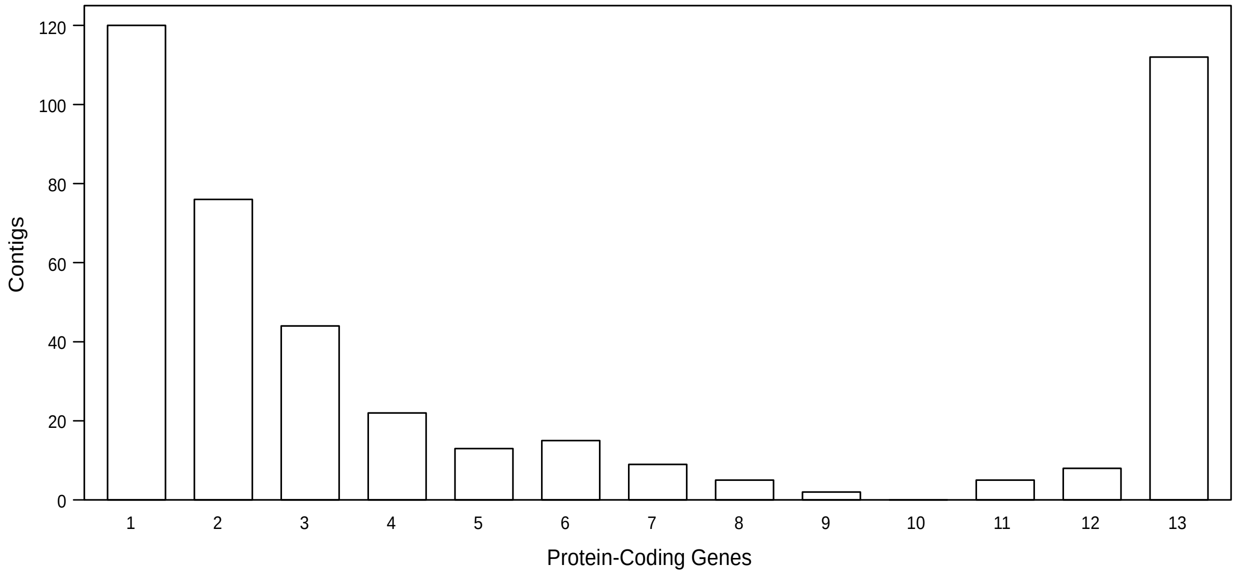

Figure S5. The mitochondrial tree-of-life for beetles under reduced taxon sampling. Maximum clade credibility consensus tree from Phylobayes analysis of 34 complete mitochondrial genomes published on GenBank (listed in Supplementary Table S2) and 124 novel 10+ protein-coding gene contigs from this study. Reference sequences are labelled with accession number and species name. Novel contigs are labelled with the most precise available taxonomic identification – species labels with numbers (and any below family-level without numbers) derive from morphospecies identifications based on bait sequences, whilst species labels with letters derive from contig position in the full beetle phylogeny (Figure 2), requiring monophyly with named reference sequences. Node labels are posterior probability values.

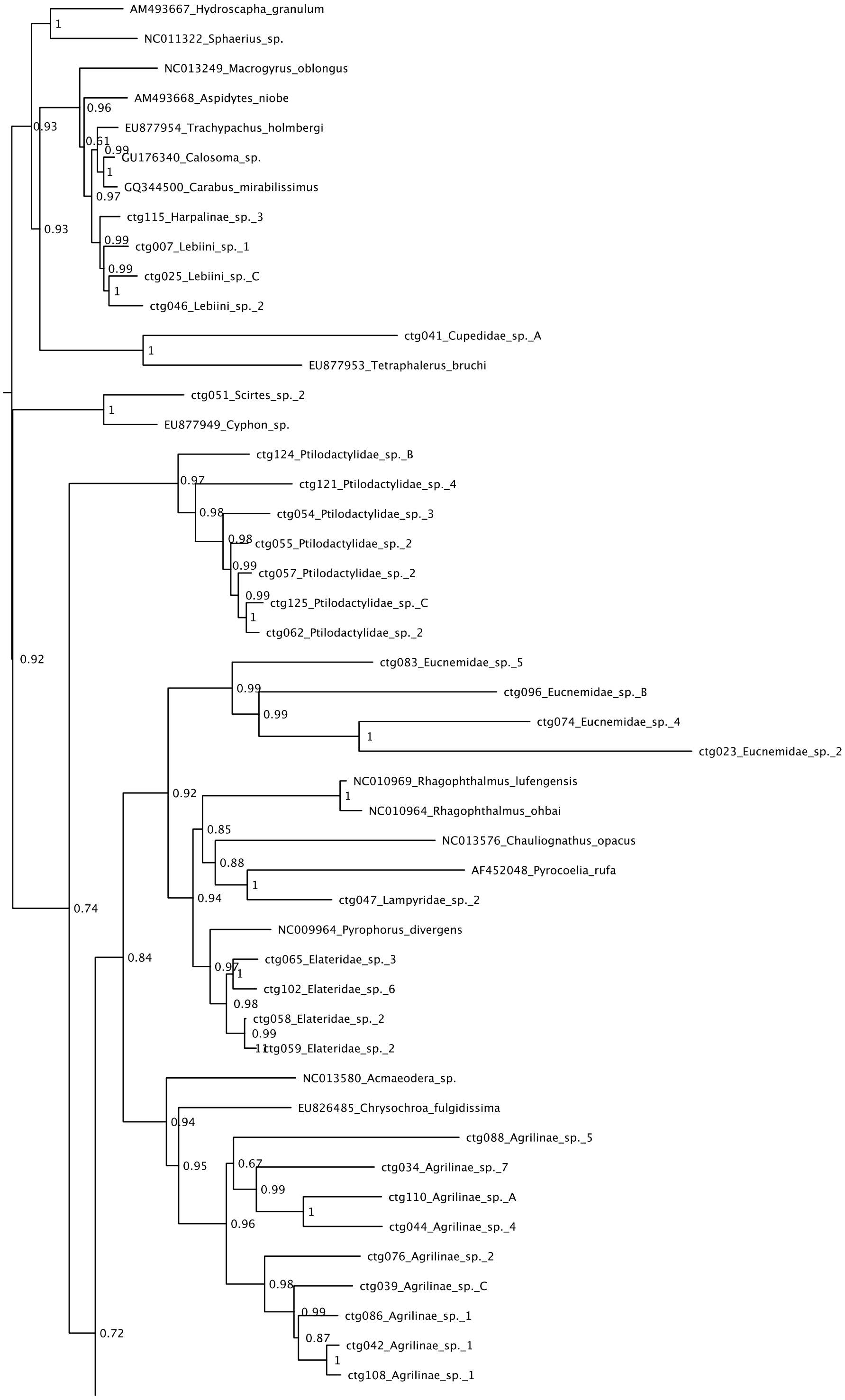

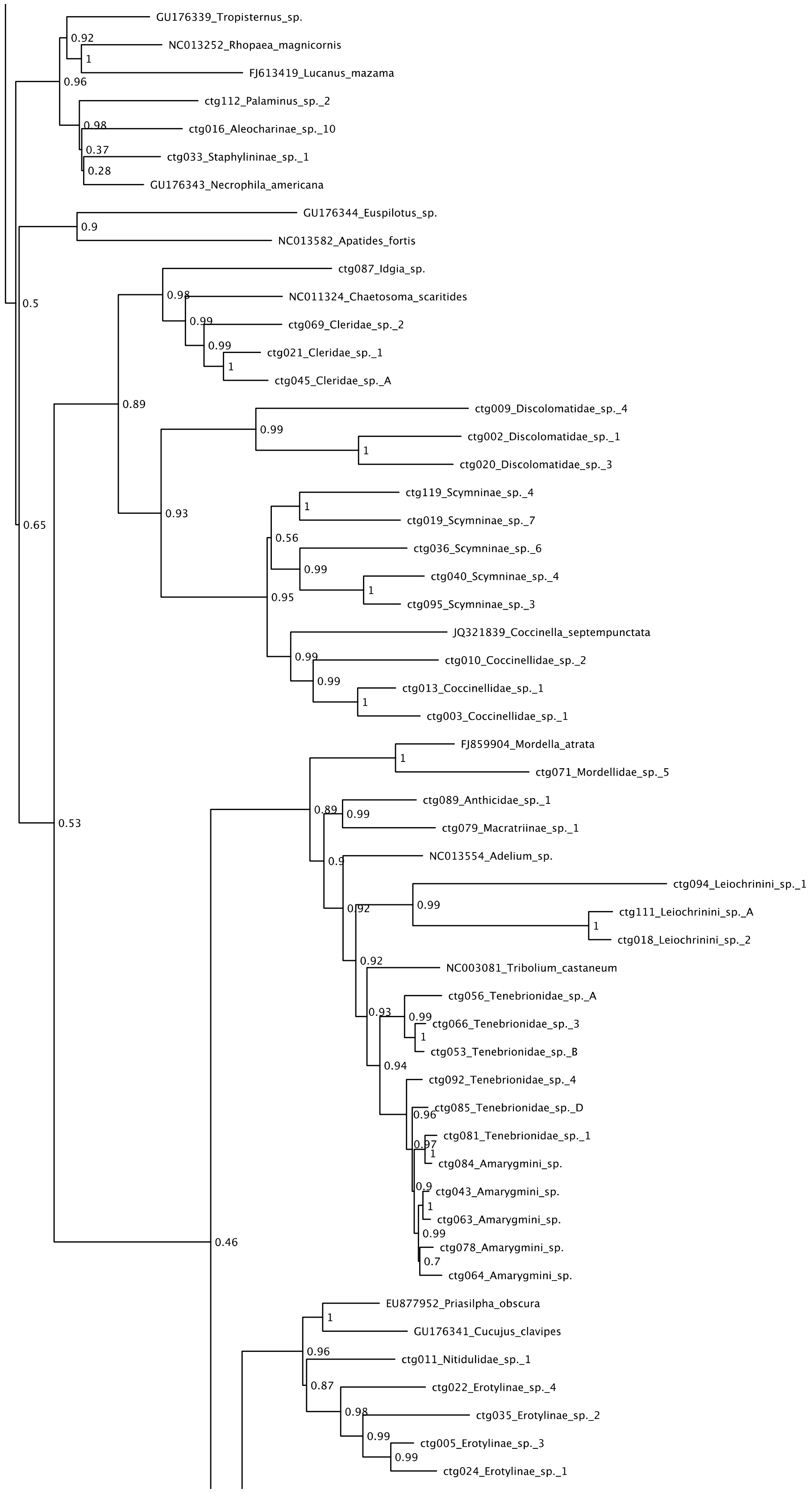
GU176339_Tropisternus_sp.
NC013252_Rhopaea_magnicornis
FJ613419_Lucanus_mazama
ctg112_Palaminus_sp._2
ctg016_Aleocharinae_sp._10
ctg033_Staphylininae_sp._1
GU176343_Necrophila_americana
GU176344_Euspilotus_sp.
NC013582_Apatides_fortis
ctg087_Idgia_sp.
NC011324_Chaetosoma_scaritides
ctg069_Cleridae_sp._2
ctg021_Cleridae_sp._1
ctg045_Cleridae_sp._A
ctg009_Discolomatidae_sp._4
ctg002_Discolomatidae_sp._1
ctg020_Discolomatidae_sp._3
ctg119_Scymninae_sp._4
ctg019_Scymninae_sp._7
ctg036_Scymninae_sp._6
ctg040_Scymninae_sp._4
ctg095_Scymninae_sp._3
JQ321839_Coccinella_septempunctata
ctg010_Coccinellidae_sp._2
ctg013_Coccinellidae_sp._1
ctg003_Coccinellidae_sp._1
FJ859904_Mordella_atrata
ctg071_Mordellidae_sp._5
ctg089_Anthicidae_sp._1
ctg079_Macratriinae_sp._1
NC013554_Adelium_sp.
ctg094_Leiochrinini_sp._1
ctg111_Leiochrinini_sp._A
ctg018_Leiochrinini_sp._2
NC003081_Tribolium_castaneum
ctg056_Tenebrionidae_sp._A
ctg066_Tenebrionidae_sp._3
ctg053_Tenebrionidae_sp._B
ctg092_Tenebrionidae_sp._4
ctg085_Tenebrionidae_sp._D
ctg081_Tenebrionidae_sp._1
ctg084_Amarygmini_sp.
ctg043_Amarygmini_sp.
ctg063_Amarygmini_sp.
ctg078_Amarygmini_sp.
ctg064_Amarygmini_sp.
EU877952_Priasilpha_obscura
GU176341_Cucujus_clavipes
ctg011_Nitidulidae_sp._1
ctg022_Erotylinae_sp._4
ctg035_Erotylinae_sp._2
ctg005_Erotylinae_sp._3
ctg024_Erotylinae_sp._1

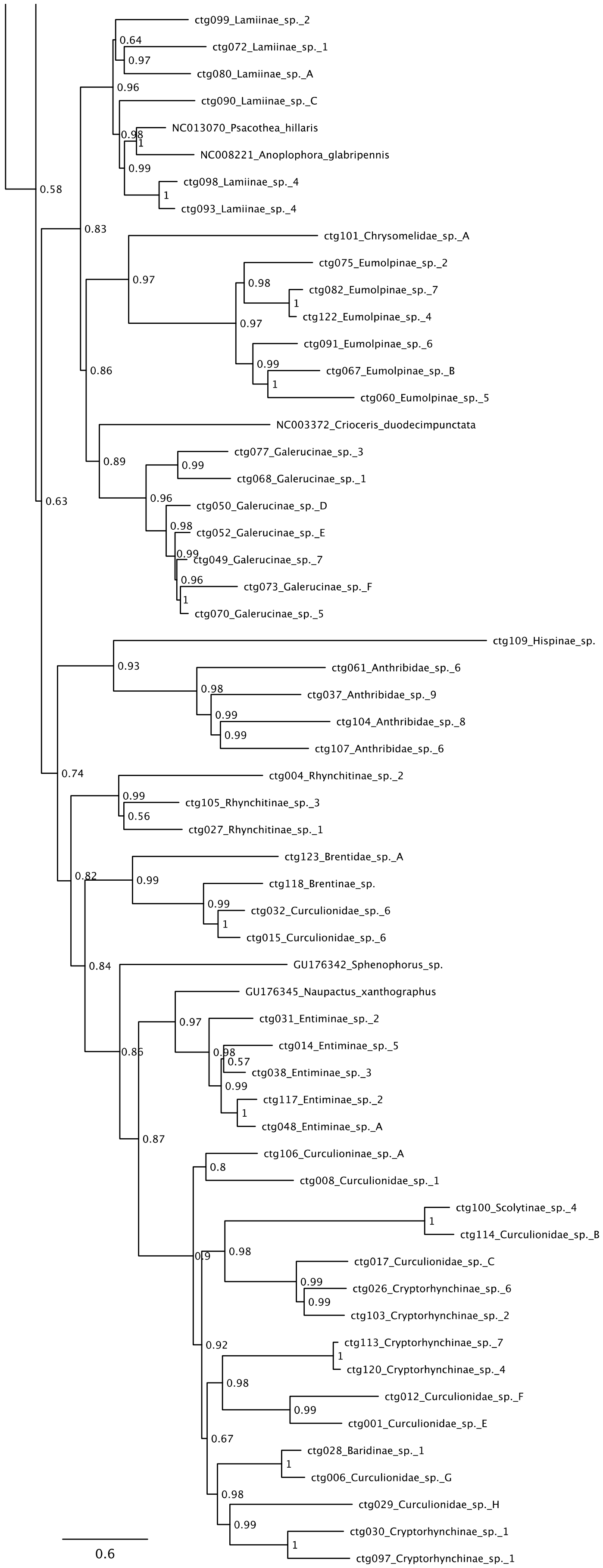
ctg099_Lamiinae_sp._2
ctg072_Lamiinae_sp._1
ctg080_Lamiinae_sp._A
ctg090_Lamiinae_sp._C
NC013070_Psacothea_hillaris
NC008221_Anoplophora_glabripennis
ctg098_Lamiinae_sp._4
ctg093_Lamiinae_sp._4
ctg101_Chrysomelidae_sp._A
ctg075_Eumolpinae_sp._2
ctg082_Eumolpinae_sp._7
ctg122_Eumolpinae_sp._4
ctg091_Eumolpinae_sp._6
ctg067_Eumolpinae_sp._B
ctg060_Eumolpinae_sp._5
NC003372_Crioceris_duodecimpunctata
ctg077_Galerucinae_sp._3
ctg068_Galerucinae_sp._1
ctg050_Galerucinae_sp._D
ctg052_Galerucinae_sp._E
ctg049_Galerucinae_sp._7
ctg073_Galerucinae_sp._F
ctg070_Galerucinae_sp._5
ctg109_Hispinae_sp.
ctg061_Anthribidae_sp._6
ctg037_Anthribidae_sp._9
ctg104_Anthribidae_sp._8
ctg107_Anthribidae_sp._6
ctg004_Rhynchitinae_sp._2
ctg105_Rhynchitinae_sp._3
ctg027_Rhynchitinae_sp._1
ctg123_Brentidae_sp._A
ctg118_Brentinae_sp.
ctg032_Curculionidae_sp._6
ctg015_Curculionidae_sp._6
GU176342_Sphenophorus_sp.
GU176345_Naupactus_xanthographus
ctg031_Entiminae_sp._2
ctg014_Entiminae_sp._5
ctg038_Entiminae_sp._3
ctg117_Entiminae_sp._2
ctg048_Entiminae_sp._A
ctg106_Curculioninae_sp._A
ctg008_Curculionidae_sp._1
ctg100_Scolytinae_sp._4
ctg114_Curculionidae_sp._B
ctg017_Curculionidae_sp._C
ctg026_Cryptorhynchinae_sp._6
ctg103_Cryptorhynchinae_sp._2
ctg113_Cryptorhynchinae_sp._7
ctg120_Cryptorhynchinae_sp._4
ctg012_Curculionidae_sp._F
ctg001_Curculionidae_sp._E
ctg028_Baridinae_sp._1
ctg006_Curculionidae_sp._G
ctg029_Curculionidae_sp._H
ctg030_Cryptorhynchinae_sp._1
ctg097_Cryptorhynchinae_sp._1
0.6

Supplementary Information S3. Failure to assign two contigs to correctly to family under requirement for monophyly with the Mito240 set.

The reliability of phylogenetic assignment relative to the fully identified Mito240 sequences was tested using the 96 10+ contigs that could be linked to specimens and family-level identification via the *cox1* barcodes. In the analysis with Mito240, 73 contigs were correctly assigned to family and a further 23 contigs to superfamily. In all but one of the latter cases, the failure to make a taxon assignment to family level was due to limited representations of small families (e.g. in Cleroidea). The exception to this was a single contig linked to Anthicidae via a bait sequence, which was placed as sister to Mycetophagidae (and therefore correctly classified as Tenebrionoidea under the requirement for strict monophyly) in spite of the formation of an otherwise monophyletic Anthicidae clade containing two reference sequences and one baited contig containing all protein-coding genes. One remaining contig could only conservatively be assigned to suborder as it was not placed within a named clade, but both its position and bait identification were consistent with assignation to the phylogenetically isolated superfamily Scirtoidea.

Supplementary Information S4. PCR primers and conditions used to amplify DNA barcodes (*cox1-5'*). For specimens that did not amplify at the first attempt (with COIF2/R2), and second attempt was made with LCO/HCO.

PCR primers

Attempt	Primer	Primer sequence 5' → 3'
1	COIF2	TCTACYAATCATAAAGATATTGGTAC
	COIR2	ACTTCTGGATGACCAAAGAATCA
2	LCO 1490	GGTCAACAAATCATAAAGATATTGG
	HCO 2198	TAAACTTCAGGGTGACCAAAAAATCA

PCR conditions with Promega GoTaq Green Master Mix

Step	Temp. (°C)	Time	No. of cycles
Initialisation	95	2 min	1
Denaturation	95	30 sec	37
Annealing	40	30 sec	
Extension	72	45 sec	
Final Extension	72	5 min	1
Final Hold	4	∞	

Table S4. Programs and settings used for mitogenome assembly.

Step	Aim	Software/process	Parameters	Additional requirements	Source
**1**	Quality assessment	FastQC 0.10.1	NA	NA	http://www.bioinformatics.babraham.ac.uk/projects/fastqc/
**2**	Adapter removal	Trimmomatic 0.30	ILLUMINACLIP:TruSeq3-PE.fa:2:30:10	TruSeq3-PE.fa (packaged with Trimmomatic)	http://www.usadellab.org/cms/index.php?page=trimmomatic
**3**	Filter for putative mitochondrial reads	BLAST 2.2.27+	-task blastn -evalue 1e-5 -max_target_seqs 1 -outfmt 6 -dust no	Mitogenome reference database for target taxon	http://blast.ncbi.nlm.nih.gov/Blast.cgi?PAGE_TYPE=BlastDocs&DOC_TYPE=Download
		Extract paired reads	NA	NA	Script 1, Supplementary Data
**4**	Assembly 1	Celera Assembler 7.0	doToggle=1 toggleUnitigLength=500 unitigger=bogart	frg file & spec file (containing parameters and path to frg file)	http://sourceforge.net/apps/mediawiki/wgs-assembler/index.php?title=Main_Page
**5**	Assembly 2	Quality control with prinseq-lite.pl	-min_len 150 -min_qual_score 20 -min_qual_mean 25 -ns_max_n 0 -trim_qual_right 20	NA	http://prinseq.sourceforge.net
		Re-pair reads	NA	NA	Script 2, Supplementary Data
		IDBA-UD	--mink 80 --maxk 230 --similar 0.98	Compile IDBA-UD to allow 250bp reads & kmers	http://i.cs.hku.hk/~alse/hkubrg/projects/idba_ud/
**6**	Filter Assemblies 1 & 2 for mitochondrial contigs	BLAST 2.2.27+	-task blastn -evalue 1e-5 -max_target_seqs 1 -outfmt 6	Mitogenome reference database for target taxon	http://blast.ncbi.nlm.nih.gov/Blast.cgi?PAGE_TYPE=BlastDocs&DOC_TYPE=Download
		Filter BLAST hits for alignment length >1kb	awk '($4>=1000){print}'	NA	NA
		Extract mitochondrial contigs with cdbfasta/cdbyank	NA	Filtered BLAST hit table from previous step	http://sourceforge.net/projects/cdbfasta/
**7**	tRNA annotation	COVE 2.4.4	NA	tRNA covariance models	http://selab.janelia.org/software.html
		Generate annotated GenBank format files	NA	NA	Script 3, Supplementary Data
**8**	Extract inter-tRNA regions	Add terminal tRNA annotations	NA	NA	Script 4, Supplmentary Data
		FeatureExtract 1.2	feature type: tRNA; extract intergenic regions	NA	http://www.cbs.dtu.dk/services/FeatureExtract/
**9**	Identify genes by mapping	Geneious 6.1	Iterate 3 times; maximum mismatches per read 50%; search more thoroughly for poor matching reads	Mitogenome reference e.g. *Tribolium castaneum* [GenBank:NC_003081]	http://www.geneious.com

Script 1. `FastqExtract.pl` – extract paired FASTQ reads based on BLAST output (i.e. filter FASTQ file using BLAST results).

Use concatenated BLAST results (tabular format) from R1 and R2 to run the script twice, once for each.

```
print "what is the name of the BLAST output file?\n";
$blastIn = <STDIN>;
chomp $blastIn;
print "what is the name of the raw reads file?\n";
$filename = <STDIN>;
chomp $filename;
print "what is the read identifier (first 6 header characters, not inc @)?\n";
$idf = <STDIN>;
chomp $idf;

#INPUT:

$fastq = $filename;
open(FASTQ, "<$fastq") or print "could not open file $fastq";

#HEADERS:

$headers = $blastreader;
chomp $headers;
open(HEADERS, "$headers") or print "could not open file $headers";
@headers = <HEADERS>;
close HEADERS;

foreach $head(@headers){
if($head =~ m/\w/){
@head = split(" ", $head);

#add to hash
$headers{$head[0]}=1}
}

$outfile = "$fastq\.out";

open(OUTFILE, ">$outfile") or print "could not open file $outfile";

########################################

open(FASTQ, "<$fastq");
while(<FASTQ>){
$line = $_;

if($flag == 1){$flag = 2; print OUTFILE $line;}
elsif($flag == 2){$flag = 3; print OUTFILE $line;}
elsif($flag == 3){$flag = 0; print OUTFILE $line;}

        elsif($line =~ m/$idf:/){
        @line = split(" ", $line);
        $line[0] =~ s/\@//ig;

                if(exists $headers{$line[0]}){
                ++$counter; $flag = 1;
                print "$counter\:\t\t$line";
                print OUTFILE "$line";
                }
        }
}
close OUTFILE;
```

Script 2. Re-pair quality controlled reads from Prinseq.

Source: http://www.bigsa.org.au/node/92 (Last accessed 02/09/2013)

Requires cdbfasta from http://sourceforge.net/projects/cdbfasta/

Where in_R1.QC.fastq and in_R2.QC.fastq are quality controlled FASTQ files from Prinseq:

```
#concatenate R1 and R2
cat in_R[12].QC.fastq > in.QC.fq

#convert IDs to older Illumina format(needed for the awk script below)
cat in.QC.fq | awk '{if (NR % 4 == 1) {split($1, arr, ":"); printf
"%s_%s:%s:%s:%s:%s#0/%s\n", arr[1], arr[3], arr[4], arr[5], arr[6], arr[7], substr($2, 1,
1), $0} else if (NR % 4 == 3){print "+"} else {print $0} }' > in.QC.nwblr.fq ;

#use cdbfasta to index the files
cdbfasta -Q in.QC.nwblr.fq

#retrive list of IDs
cdbyank in.QC.nwblr.fq.cidx -l > in.QC.ids

#retrieve list of IDs for paired reads
awk -v sep="/" '{ if ((sep_i=index($0,sep)) > 0) { name=substr($0,1,sep_i-1);
suffix=substr($0,sep_i); } else { name=$0; } if (r[name]) { print name r[name]; print $0;
delete r[name]; } else { r[name]=suffix; }}' in.QC.ids > in.QC.paired.ids

#make an interleaved FASTQ file for paired reads
cdbyank in.QC.nwblr.fq.cidx < in.QC.paired.ids > in.QC.paired.fq
```

Script 3. `cove_output.pl` – generate tRNA annotated GenBank format files from COVE output files.

```
use Bio::SeqIO;

#Remove some (old) files first
@tempfiles = glob("*.tmp");
foreach $tempfile(@tempfiles){unlink $tempfile;}

#The COVE output files to process
@files = glob("*.out");

#Directory to store resulting GenBank formatted output files
mkdir(GB);

foreach $file(@files){
       open(FILE, "$file") or print "could not open $file";
       @data = <FILE>;
       close FILE;

foreach $line(@data){

#new model -> hash,sort and start again
                    if($line =~ m/Model/){
                           @allKeys = keys(%hash);
                           @allKeys = sort{ $a <=> $b} @allKeys;

#select one with highest value, but only if above ##40##
                           $highest = pop(@allKeys);
                           $value = $hash{$highest}; if($highest > 40){
                           $order{$value} = ("$model\t$highest");
                    }

#select penultimate one for Ser and Leu ##two tRNAs for each##
                    if($model =~ m/Leu/){
                           $highest = pop(@allKeys);
                           $value = $hash{$highest}; if($highest > 40){
                           print "$model\t$highest\t$value\n";
                           $order{$value} = ("$model\t$highest");}
                    }
                    if($model =~ m/Ser/){
                           $highest = pop(@allKeys);
                           $value = $hash{$highest}; if($highest > 40){
                           $order{$value} = ("$model\t$highest");}
                    }
                    %hash = "";
                    @line = split("\:", $line);
                    $model = $line[1]; chomp $model;
                    print "$model\n";
             }
             elsif($line =~ m/version/){next;}
             elsif(($line =~ m/^ /) || ($line =~ m/^1/)){
                    $line =~ s/^1/^ 1/ig; #waarden, vul de hash
                    ++$counter; @line = split(" {1,8}", $line);

#tRNA positions, add counter to make unique
                    $position = "$line[2]\_$line[3]\_$counter";
       $hash{"$line[1]"} = "$position";
             }
             else{next;}
       }
             @allKeys = keys(%hash);
             @allKeys = sort{ $a <=> $b} @allKeys;
             $highest = pop(@allKeys);
             $value = $hash{$highest}; if($highest > 40){
             $order{$value} = ("$model\t$highest");}
             %hash = ();
             @allKeys = keys(%order);
             @allKeys = sort{ $a <=> $b} @allKeys;
             $file2 = $file;
```

```
              $file2 =~ s/out/tmp/ig; #temporary file
              $file3 = $file;
              $file3 =~ s/.out//ig; #base name of genbank flat file

#change to GenBank flatfile format
              $seq_in = Bio::SeqIO->new('-file' => "$file3", '-format' => "fasta");
              $seq_out = Bio::SeqIO->new('-file' => ">>$file2", '-format' => "genbank");
              while($inseq = $seq_in->next_seq){$seq_out->write_seq($inseq);
              open(OUTFILE, ">>GB\/$file3.gb");

open(FILE, $file2);
@data = <FILE>;
close FILE;

#Add the tRNA annotations to the GenBank flatfile format
foreach $line(@data){
if($line =~ m/FEATURES/){ print $line; print OUTFILE $line;

              foreach $key(@allKeys){
                            if($key == "_"){next;}else{
                            @key = split("_", $key); $trna = $order{$key}; $trna =~ s/
//ig; @trna = split('.cm', $trna);

                            if($key[0]<$key[1]){print OUTFILE "     tRNA
$key[0]..$key[1]\n                      \/gene=\"$trna[0]\"\n";}

if($key[1]<$key[0]){print OUTFILE "     tRNA            complement\($key[1]..$key[0]\)\n
\/gene=\"$trna[0]\"\n";}

}}}else{print OUTFILE $line;}}

close OUTFILE;

#

system("dos2unix -u GB\/$file3.gb -o");

              %order = ();
              $counter = 0;
}
```

Script 4. `add_start_stop.pl` – add a 1bp tRNA annotation to the start and end of each GenBank formatted sequence.

```
@files = glob("*.gb");
foreach $file(@files){
open(FILE, "$file");
@data = <FILE>;
close FILE;

$file =~ s/gb/genbank/ig;
open(OUTFILE, ">$file");

foreach $line(@data){

if($line =~ m/LOCUS/){
print OUTFILE $line;
@line = split(" ", $line);
$end = ($line[2]);}

elsif($line =~ m/FEATURES/){
print OUTFILE $line;

print OUTFILE "     tRNA            1..1\n                     \/gene=\"startmarker\"\n";
print OUTFILE "     tRNA            $end..$end\n
\/gene=\"endmarker\"\n";

$end = 0;}

else{print OUTFILE $line;}

}
}
```
